# CD34^+^CLDN5^+^ tumor associated senescent endothelial cells through IGF2-IGF2R signaling increased cholangiocellular phenotype in hepatocellular carcinoma

**DOI:** 10.1016/j.jare.2024.12.008

**Published:** 2024-12-12

**Authors:** Xin-yu Zhu, Wen-ting Liu, Xiao-juan Hou, Chen Zong, Wei Yu, Zhe-min Shen, Shu-ping Qu, Min Tao, Meng-meng Xue, Dao-yu Zhou, Hao-ran Bai, Lu Gao, Jing-hua Jiang, Qiu-dong Zhao, Li-xin Wei, Xue Yang, Zhi-peng Han, Li Zhang

**Affiliations:** a Changhai Clinical Research Unit, Changhai Hospital of Naval Medical University, Shanghai, China; b Tumor Immunology and Metabolism Center, National Center for Liver Cancer, Naval Medical University, Shanghai, China; c Department of Hepatic Surgery, Third Affiliated Hospital of Naval Medical University, Shanghai, China; d Department of Clinical Pharmacology, The Second Hospital of Anhui Medical University, Hefei, China; e Department of Oncology, Longhua Hospital Affiliated to Shanghai University of Traditional Chinese Medicine, Shanghai, China; f Department of Medical Oncology, Fudan University Shanghai Cancer Center, Shanghai, China

**Keywords:** Single-cell RNA sequencing, Tumor associated senescent endothelial cells, Cholangiocellular phenotype, Mesenchymal stem cells

## Abstract

**Introduction:**

The heterogeneity of hepatocellular carcinoma (HCC) is linked to tumor malignancy and poor prognosis. Nevertheless, the precise mechanisms underlying the development of the cholangiocellular phenotype (CCA) within HCC remain unclear. Emerging studies support that the cross-talk among the host cells within tumor microenvironment (TME) sustains the cancer cell plasticity.

**Objectives:**

This study sought to identify the specific cell types involved in the formation of CCA and to elucidate their functional roles in the progression of HCC.

**Methods:**

**S**ingle-cell RNA sequencing was employed to identify the specific cell types involved in the formation of CCA. Both in vitro and vivo analyses were used to identify the tumor-associated senescent ECs and investigate the function in TME. The diethylnitrosamine-induced model was utilized to investigate the interaction between senescent ECs and MSCs, aiming to elucidate their synergistic contributions to the progression of CCA.

**Results:**

Using single-cell RNA sequencing, we identified a distinct senescent-associated subset of endothelial cells (ECs), namely CD34^+^CLDN5^+^ ECs, which mainly enriched in tumor tissue. Further, the senescent ECs were observed to secrete IGF2, which recruited mesenchymal stem cells (MSCs) into the TME through IGF2R/MAPK signaling. In primary liver cancer model, MSCs exhibited a strong tumor-promoting effect, increasing the CCA and tumor malignancy after HCC formation. Interestingly, knockdown of IGF2R expression in MSCs inhibited the increase of CCA caused by MSCs in HCC. Meanwhile, it was revealed that MSCs released multiple inflammatory and trophic-related cytokines to enhance the cancer stem cell-like characteristics in HCC cells. Finally, we demonstrated that CEBPβ up-regulated IGF2 expression in tumor senescent ECs by combining with *Igf2*-promtor-sequence.

**Conclusions:**

Together, our findings illustrated that tumor associated senescent ECs in HCC recruited the MSCs into TME, enhancing cancer stem cell (CSC)-like features of HCC cells and contributing to the CCA formation.

## Highlights

• Utilizing single-cell RNA sequencing technology, we have identified a distinct subset of tumor-associated senescent endothelial cells.
• This study demonstrated that senescent endothelial cells increased the cholangiocellular phenotype within hepatocellular carcinoma.
• This study reveals that the cross-talk between the senescent endothelial cells and mesenchymal stem cells fostered the tumor progression.
• Senescent endothelial cells recruited mesenchymal stem cells into the tumor microenvironment via IGF2-IGF2R signaling.

## Graphical abstract

**Figure:**
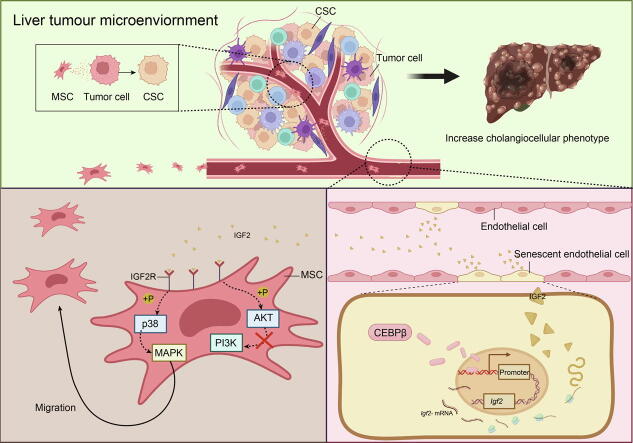

## Introduction

Globally, liver cancer is the sixth most common cancer and the third leading cause of death in cancer[1], [2].The prognosis for liver cancer is relatively poor, with the five-year relative survival rate for primary liver cancer ranging from only 5 % to 30 %[3]. Clinically, numerous factors, primarily associated with the stage at diagnosis, treatment methods, and tumor heterogeneity, influence the prognosis of liver cancer. In terms of therapeutic methods, surgical resection, liver transplantation, and local ablation therapies offer the possibility of cure, especially for early-stage liver cancer patients. Transarterial chemoembolization, stereotactic body radiotherapy, endovascular therapy and systemic treatments (such as targeted therapies and immunotherapies) provide opportunities for extending survival and improving the quality of life for patients with advanced liver cancer[4], [5]. Regarding the heterogeneity of liver cancer, hepatocellular carcinoma (HCC) is the most common type with a relatively better prognosis, accounting for approximately 80 % of all liver cancer cases; combined hepatocellular carcinoma-cholangiocarcinoma (cHCC-CCA) and other rare types of liver cancer have a prognosis that is the next best, accounting for about 5 % of all cases; intrahepatic cholangiocarcinoma (iCCA) has the worst prognosis, accounting for about 15 % of all cases[6]. The cholangiocellular phenotype (CCA) in liver cancer denotes a distinct subtype of HCC that presents with traits of cholangiocytes. This implies that HCC, in certain cases, can express markers that are traditionally linked to cholangiocytes, including CK19 and EPCAM. When HCC presents with these cholangiocyte features, it is often referred to as cholangiocarcinoma-like hepatocellular carcinoma[7]. Recent studies evidenced that the dual-phenotype hepatocellular carcinoma and CK7-CK19 positive cHCC-CCA were much more aggressive than the HCC. Moreover, the formation of CCA within HCC prompts a poorer prognosis in patients [8], [9]. However, the specific mechanism by which the CCA within HCC arises remains unclear.

Recent research supports the idea that CCA formation was closely related to the cross-talk between parenchymal cells (such as hepatocytes and cholangiocytes) and non-parenchymal cells (including fibroblasts, stellate cells, Kupffer cells, and endothelial cells) in tumor microenvironment (TME)[10]. These discrete cell types release a series of signals that translate into a pathological entity, thereby maintaining the cancer cell plasticity, invasiveness, and migration abilities[11]. Most past studies have focused on the interaction between immune cells and cancer cells. In fact, the cross-talk between the interstitial cells also plays an significant role in contribution to the HCC heterogeneity.

Notably, endothelial cells (ECs) as a member in TME participate in angiogenesis, regulating the proteins, oxygen, cytokines, nutrition, and cells into the tumor site. Emerging evidences have indicated that the ECs in liver secreted adhesion molecules (such as ICAM1) and immunosuppressive factors (such as PDL1) to recruit immune cells (such as macrophage) or tumorigenic cells into TME, inhibiting and limiting the host cellular anti-tumor activity[12], [13], [14]. Generally, ECs in HCC tissues and normal tissues exhibit heterogeneity and have both molecular and functional differences[15]. An scRNA-seq study focusing on EC in human HCC identified 11 different EC clusters, and it was predicted that three of these ECs (IGFBP3^+^ECs, PLVAP^+^ECs and PLPP3^+^ECs) participated in constructing the TME[16]. However, due to the lack of specific markers to monitor and distinguish tumor-associated ECs, research on the physiological roles of ECs in TME is progressing slowly. Similarly, the underlying mechanism of the tumor-promoting effect of ECs is uncompleted known.

In summary, the aim of this paper is to identify the specific cell types involved in the formation of tumor heterogeneity and to elucidate their functional roles in liver cancer. Here, we employed single cell RNA sequencing (sc-RNA seq) technology to analyze heterogeneity of ECs during HCC through the diethylnitrosamine (DEN)-induced model. We identified a distinct subset of tumor associated senescent ECs, which were present in tumor tissue and promoted the development of CCA within HCC. In addition, our findings uncovered a significant crosstalk between tumor associated senescent ECs and mesenchymal stem cells (MSCs), resulting in increase of CCA within HCC. Furthermore, we performed proteomic analysis and gene editing technology, as well in vitro and vivo experiments, to uncover the molecular mechanisms behind the promotion of tumor progression by tumor-associated senescent ECs. Our results illustrate the distinct role of senescent ECs in HCC and highlight the interaction among cancer cells, MSCs, and ECs. The discoveries will facilitate a better understanding of the increased heterogeneity and malignancy in HCC and provide new ideas and theoretical foundations for clinical prognosis judgments in liver cancer.

## Material and methods

### Ethics statement

All experiments were conducted complied with the ethical policies approved by Naval Medical University Animal Care Committee (Number: 20175001123) and the Ethics Committee of the Third Affiliated Hospital of Naval Medical University (Number: EHBHKY2017-K-025).

### Animals models

Healthy male Sprague-Dawley rats (2- to 3- month-old, weighing 200–300 g) were obtained from Jihui Laboratory Animal Care Co., Ltd. (Certificate 20220009008147). The animals were kept in the SPF animal room. After a one-week acclimation period in the SPF facility, the rats were blindly randomly divided into 3–4 groups, with 3–6 rats in each group. 0.1 % DEN (Sigma-Aldrich, St. Louis, MO, USA) in drinking water was used to establish the primary liver cancer rat model. After 12 weeks, CD34^+^CLDN5^+^ ECs (10^6^ cells/200 μl saline solution), CD34^−^CLDN5^−^ ECs (10^6^ cells/200 μl saline solution), the MSCs (10^6^ cells/200 μl saline solution), the silent IGF2R MSCs suspensions (10^6^ cells/200 μl saline solution) and PBS were injected via spleen or tail vein into the DEN-treated rats. Liver tissues and blood samples were collected at indicated time for biological analysis. Meanwhile, the serum was separated from the blood to determine the levels of ALT and AST. All animal experiments and protocols were carried out in strict accordance with the relevant regulations of experimental animals, and approved by Naval Medical University Animal Care Committee.

### Patients and tissue samples

Specimens of liver tissue were obtained from 68 patients with primary liver cancer who underwent hepatic resection at the Third Affiliated Hospital of Naval Medical University, including 48 men and 20 women. The median age of the patients was 56.38 (Age range: 20–76 years). Details of the patients were shown in Table S1. The 66 patients' specimens were subjected to IHC analysis. Flow cytometry was performed on tumor tissue and *peri*-tumor tissue from 2 patients. Prior informed consent was obtained. Then, the study protocol was approved by the Ethics Committee of the Third Affiliated Hospital of Naval Medical University.

### Library construction and sequencing

To create a single-cell library, the liver tissues, cancerous tissues and *peri*-cancerous tissues as samples from D4, D8, D12 and D16 DEN-pretreated rats were used to perform single-cell RNA sequencing technology according to the Chromium Single Cell 3ʹ Reagent Kits v3 (10 × Genomics) manufacturer's instructions. Briefly, the liver tissues were mechanically minced and enzymatically digested using an enzymatic cocktail containing 60 ml dispase (5 U/ml, Stemcell Technologies), 900 µl liberase (1 g/l, Sigma Aldrich), 0.03 g DNase (Sigma Aldrich) at 37 °C, 45 min. GEM generation, barcoding, and cDNA library preparation were completed by the CCHMC Gene Expression Core using the Chromium Single Cell 3′ Reagent Kit (10 × Genomics version 2.0). Sequencing was performed on NovaSeq 6000 at a depth of 300–450 million reads. Read alignment and gene-expression quantification of data were performed using the CellRanger pipeline (10 × Genomics version 2.1.1). Rat scRNA-seq data was aligned to rat genome (ensemble v93). Cells with at least 500 expressed genes (UMI > 0) and less than 25 % of UMIs mapping to mitochondrial genes were included for downstream analysis. Datasets were integrated using harmony, and downstream analyses were performed in R (version 3.6.1) using Seurat (v 2.3.4). Significant genes (FDR ≤ 1e − 3) were selected for principal component analysis (PCA).We selected unsupervised cell clusters of the same major cell type for t-distributed stochastic neighbor embedding and uniform manifold approximation and projection (UMAP) analysis. Clusters were computed using the FindClusters function (resolution = 0.6). Cell types were defined according to marker genes. Genes with *p* value < 0.05 and effect size > 2 expressed in > 20 % of the cells in each cluster were considered significant. We used the R package SCENIC (1.3.1), RcisTarget (1.12.0), and AUCell (1.14.0) to analyze the enrichment of transcriptome factors in cell subsets.

### Isolation of primary MSCs

The tibia and femur bone marrow aspirates from wild-type male rats (2- to 3- month-old, weighing 200–250 g) were used to obtain the MSCs. The bone marrow aspirates were cultured in complete DMEM medium supplemented with 10 % FBS, 1 % penicillin–streptomycin, 1 % GlutaMAX^TM^-I (Gibco, Grand Island, NY, USA), 10 ng/ml rat basic fibroblast growth factor (PeproTech,Cranbury, NJ,US) in tissue culture flasks. After 72 h, the non-adherent cells were removed with fresh DMEM medium, and replaced the medium every 3 days. Cells were characterized according to our previous study[17].

### Isolation of primary CD34^+^CLDN5^+^ ECs

The liver tissues from 16 weeks DEN-pretreated rat were used to obtain the ECs. The cells were isolated and cultivated by following the protocol[18]. 0.05 % Collagenase Type Ⅳ was utilized to digest the liver. After differential centrifugation, the cellular suspension was layered on top of a two-step Percoll gradient (20 ml 25 % Percoll on the top and 15 ml 50 % Percoll on the bottom, GE HealthCare, Chicago, IL, US) and centrifuged at 900 g for 20 min. 15- to 20- ml fluid from the tube was enriched in ECs. Then, ECs suspension was collected and markered by combination of CD34 (Novus) and CLDN5 (Bioss) antibodies for fluorescence-activated cell sorting (FACS). The Cell Sorter (Beckman Counlter, MoFlo Astrios^EQ^,US) was used to sort ECs. The CD34^+^CLDN5^+^ ECs and CD34^−^CLDN5^−^ ECs that were sorted out were cultured in complete RPMI 1640 medium supplemented with 20 % FBS and 1 % penicillin–streptomycin (Gibco, Grand Island, NY, USA) in cellular culture flasks. After 48 h, the non-adherent cells were removed by fresh medium and replaced the medium every 2 days. After 5- to 6- days, the ECs were used for experiment. CD34 antibody (Servicebio, Wuhan, CN) was utilized to characterize the cells by immunocytofluorimetric assay.

### Flow cytometry

Briefly, tumor tissue and *peri*-tumor tissue from HCC patients were digested at 37 °C for 30 min with 1 mg/mL Collagenase Type Ⅳ (Gibco, Grand Island, NY, USA). Digestion was stopped by adding RPMI 1640 medium (Gibco, Grand Island, NY, USA) and the cells were filtrated through 100 μm cell strainers. The cell suspension was centrifuged at 100 g for 5 min to remove most of the parenchymal cells. Then, the supernatant, enriched in ECs, was centrifuged for 7 min at 650 g. The cells were resuspended and stained with following antibodies on ice for 30 min: anti-human CD45 (BD Pharmingen), anti-human CD34 (Biolegend), anti-human/rat CLDN5 (Bioss). For intracellular staining, cells were resuspended in fix/perm buffer (Biolegend,San Diego, CA, US) on ice for 30 min, and then washed twice with Intracellular Staining Perm Wash Buffer (Biolegend,San Diego, CA, US). Antibodies against human/rat P16 (SantaCruz) and P21 (SantaCruz) were added and incubated for 30 min on ice. The cytokine producing cells were determined by flow cytometry. The flow cytometry data were collected on Fortessa (BD) and analyzed using FlowJo (Tree Star). Similarly, the primary hepatic ECs from D16 rat tumor tissue were stained by anti-rat CD45 (BD Pharmingen), anti-rat CD34 (Novus), anti-human/rat CLDN5 (Bioss), anti-human/rat P16 and anti-human/rat P21(SantaCruz) for detection by flow cytometry.

### Multiplex immunohistochemistry (mIHC) and immunofluorescence

Rats were sacrificed and transcardially perfused with phosphate buffered saline and 4 % paraformaldehyde. Liver tissues were harvested for pathological assessment. Paraffin-embedded livers were sectioned into 5 µm sections and stained with hematoxylin-eosin according to the manufacturer's protocol. For immunohisochemistry, primary antibodies including anti-CD146 (1:250, Abcam), anti-CD90 (1:250, Abcam), anti-CD73 (1:200, Abcam), anti-CK7 (1:1000, Abcam), anti-CK19 (1:400, Abcam), anti-CD34 (1:500, Servicebio), anti-CLDN5 (1:200, Affinit), anti-CD44 (1:500, Abcam) and anti-EPCAM.(1:100, Abcam) were used. For immunofluroescence (IF), primary antibodies including anti-CD34 (1:500, Servicebio), anti-CLDN5 (1:250, Affinit), anti-CD146 (1:250, Abcam), anti-PDGFRβ (1:500, Abcam), anti-IGF2BP1 (1:1500, Servicebio), anti-IGF2R (1:200, Proteintech), anti-CK7 (1:1000, Abcam), anti-CK19 (1:400, Abcam), anti-p21 Cip1 (1:250, Affinit), anti-p16INK4a (1:50, Abgent), anti-γH2AX (1:250, Abcam), anti-CEBPβ (1:100, Invitrogen) were used. Then, the iF546-Tyramide, iF594-Tyramide, iF488-Tyramide and iF555-Tyramide (Servicebio, Wuhan, CN) were used as secondary antibodies. Nuclei were counterstained with 4,6-diamidino-2-phenylindole (DAPI, Servicebio, Wuhan, CN). The details of method followed according to our previous study[19].

### Gene silencing mediated by siRNA

The three or two siRNA candidates for each target (*Cebpb* and *Igf2r*) sequence were designed and listed in Table S2 and Table S3. Scrambled siRNA was used as the negative control. CY3-siRNA was utilized to determine the transfection efficacy. All reagents were obtained from Hanbio Co., Ltd (Shanghai, China) and followed the manufacturer's protocol. Briefly, the 20 µM siRNA was diluted to 100 nM with the medium. Then, the RNA transfection mixture was added to form the transfection complexes and incubated 15 min. The MSCs and ECs medium was replaced with fresh medium containing the siRNA to perform the transfection.. After 48 h, whether the targets gene pull-down was determined by the Real-time PCR (RT-PCR) and Western Blot (WB).

### Cell co-culture

About 10^3^ per well RH35 tumor cells were seeded into the lower compartment of transwell plates. About 5 × 10^4^ CD34^+^CLDN5^+^ ECs or CD34^−^CLDN5^−^ ECs per well were seeded into upper chamber of transwell plates (CORNING, Kennebunk, ME, USA). After 6 days, the RH-35 was used to perform colony and sphere formation assays. About 10^5^ MSCs seeded into the lower compartment of transwell plates and 5 × 10^4^- CD34^+^CLDN5^+^ ECs or CD34^−^CLDN5^−^ ECs per well were seeded into upper chamber of transwell plates. After 48 h, the MSCs were used to perform wound healing assays.

### Colony and sphere formation assays

MSC CM was obtained from the cell culture flasks. Before collection the MSCs were starved in DMEN medium without FBS and PS for 16 h. The supernatant was collected and centrifuged at 1000 × g, 5 min. About 10^3^- per well RH35 were seeded into a six-well plate and low attachment surface six-well plate (CORNING Kennebunk ME USA). Half of wells were replaced with the MSC-CM containing 10 % FBS and 40 % normal DMEM and while the others were replaced with DMEM containing 10 % FBS. For colony formation assay the cells were incubated at 37℃.

5 % CO_2_ for 5 days. 0.1 % crystal violet solution was used to stain. Recorded the number of the colony (>50 cells). For sphere formation assays the cells were incubated at 37 ℃ 5 % CO_2_ for 5 days which the number of colonies (greater than 50 cells) was recorded under a confocal microscope (Leica TCS SP2 Germany).

### Wound healing and transwell assays

About 10^5^ MSCs were seeded into the 24-well plates. 5 × 10^4^- to 1 × 10^4^- per well MSCs were seeded into transwell plates (CORNING, Kennebunk, ME, USA). For wound healing assays, the cells were incubated for 24 h and disrupted by scratching with a 10 µl microsterile pipette tips. 500 µl condition mediums were replaced in each well. The cells photographed at 0 h, 24 h and 48 h by phase-contrast microscope. For transwell assays, the condition medium with 5 × 10^4^- CD34^+^CLDN5^+^ ECs/5 × 10^4^- CD34^−^CLDN5^−^ ECs/IGF2 was added into the lower compartment and the MSCs with DMEM medium were added into the upper chamber. After 48 h, the MSCs that had migrated to the lower surface of the membrane were fixed with 4 % paraformaldehyde and stained with a 0.1 % crystal violet solution. Photographed using the phase-contrast microscope. Image-Pro Plus software (Version 6.2, Media Cybernetics, Bethesda, MD, USA) was utilized to calculate the area of wounding and the number of the MSCs.

### Cleavage under targets and tagmentation (CUT&Tag)

First of all, the JASPAR 2022 database[20] was used to predict sequence of the CEBPβ transcription factor binding sites within *Igf2* promoter. The three candidates sequence were listed in Table S4 and were obtained from Generay Biotech Co., Ltd (Shanghai, China). The CUT&Tag technology was used to verify that the CEBPβ bound the promoter of the IGF2 in ECs. Most of the reagents were obtained from NovoNGS®CUT&Tag 3.0 High-Sensitivity kit (Novoprotein, Shanghai, CN). The procedure followed the manufacturer's instructions. Briefly, the CD34^+^CLDN5^+^ ECs were incubated with the ConA Beads for 1 h. The primary antibodies including CEBPβ (1:50, Invitrogen) and normal rat IgG (1:50, Santa Cruz Biotechnology) was combined with the samples for 2 h at room temperature. Then the goat-anti-rabbit secondary antibody (1:5000, Bioworld) and goat-anti-rat secondary antibody (1:5000, Bioworld) were incubated with the samples for 1.5 h. The samples were bound with the ChiTag transposon and fragmented using Tragmentation Buffer in PCR instrument. The Tagment DNA extract beads were utilized to extract the DNA from the samples and performed the PCR amplification (PCR condition were used: 72 ℃ for 3 min, 98 ℃ for 30 s, followed by 25 cycles of 98 ℃ for 15 s, 60 ℃ for 10 s, 72 ℃ for 8 s). After PCR productions purification, the Real-time PCR was performed in a total reaction volume of 20 µl (10 µl SYBR green, 1 µl forward and reverse specific primers, respectively, 1 µl complementary DNA and 8 µl ddH_2_O), PCR conditions: 95 ℃ for 10 min, followed by 40 cycles of 95 ℃ for 15 s, 60 ℃ for 30 s, and 72 ℃ for 30 s.

### Western blot and gel electrophoresis

For western blot assays, the samples were lysed by the Radio Immunoprecipitation Assay Lysis Buffer. Then, 10–25 µg protein samples were electrophoresed by SDS-PAGE (Bio-Rad, Hercules, CA, USA) with 4–20 % Bis-Tris SurePAGE gel (GenScript, Nanjing, CN). Polyvinyl difluorid membranes (Merck Millipore, Darmstadt, Germany) were used to transfer the proteins. Next, the membranes were incubated with the primary antibodies including anti-SOX2 (1:500, Affinit), anti-NANOG (1:500, Affinit), anti-OCT3 (1:1500, Afinit), anti-AKT (1:1000, CST), anti-p-AKT (1:1000, CST), anti-IGF2R (1:1000, Proteintech), anti-p-p38 MAPK (1:1000, CST), anti-p38 MAPK (1:1000, Affinit), anti-GAPDH (1:5000, Bioworld) at 4 ℃ for overnight, which followed by incubation with secondary antibody for 1.5 h. The membranes were visualized using enhanced chemiluminescence detection reagents (GE HealthCare, Chicago, IL, US). For gel electrophoresis, 1–5 μg of DNA samples were loaded to a 2 % agarose gel, followed electrophoresis at 160 V for 30 min in TBE buffer. The gel was stained with ethidium bromide staining and visualized by gel imaging system (SYNGENE, Frederick, MD, UK).

### Real-time PCR

Total RNA was extracted using Trizol Reagent, which was obtained from Nanjing Vazyme BioTech Co., Ltd. The operation was followed by the manufacturer's instruction. Bestar™ qPCR RT Kit (DBI, Germany) was utilized to produce the complementary DNA. The LightCycler® 96 Real-time PCR (Roche, USA) was used to perform the PCR in a total reaction volume of 20 µl (10 µl SYBR Green, 1 µl each of forward and reverse specific primers, 1 µl complementary DNA, and 8 µl ddH_2_O). PCR conditions: 95 ℃ for 10 min, followed by 40 cycles of 95 ℃ for 15 s, 60 ℃ for 30 s, and 72 ℃ for 30 s. The primer information for *Sox2*, *Nanog, Oct3, Igf2r, Igf1r, Insr* and *Gapdh* were shown in Table S5.

### Olink proximity extension assay

The Olink Proximity Extension Assay (Olink Proteomics AB, Uppsala, Sweden) was used to perform proteome analysis between MSCs-CM and normal medium. The MSCs-CM and normal medium cargo were analysed using the Olink Inflammatory and oncology panels. Olink platform is a multiplex DNA-coupled immunoassay-based targeted proteomic approach in which each target protein is detected by a pair of unique oligonucleotide-labeled antibodies. When the antibody probes bind to their target protein, form a target sequence that is later quantified by RT-PCR. Counts of known sequences are thereafter translated into normalized protein expression (NPX, which is an arbitrary unit on a log2-scale where a high value corresponds to a higher protein expression) units through a quality control and normalization process developed and provided by Olink. Data were quality controlled and normalized using an internal extension control and an inter-plate control, to adjust for intra- and inter-run variation.

### Electrophoretic mobility shift assays (EMSA)

The coding sequences of *Cebpb* were amplified and cloned into pET30a vector (digested with BamH I and Kpn I). His-CEBPβ fusion protein were expressed in *E.coli* strain Rosetta (DE3). The fusion protein was induced by 0.2 mM IPTG and purified by Nickel Agarose Magnetic Beads (Ni-NTA Agarose, QIAGEN, Cat No.30230, Germany). Complete the EMSA experiment according to the Chemiluminescent EMSA Kit instructions (Thermo Scientific, United States).

### Statistical analysis

All experiments were performed at least three times. Analysis of variance was performed using GraphPad Prism 9.0 (GraphPad Software). Quantitative data were expressed as mean ± SD, for each experiment. Significance between groups was performed using Student's *t* test. Clinical data analysis was performed using SPSS 20.0 for Windows (SPSS Inc., Chicago, IL, USA); Pearson's correlation coefficient was used to determine correlations between continuous normally distributed variables. Kaplan-Meier analysis was used to determine the survival duration. Log-rank test was used to compare patients' survival duration time between each group. Statistical significances are indicated by *p < 0.05, **p < 0.01, ***p < 0.001, ****p < 0.0001.

## Results

### scRNA-seq analysis revealed a senescence − associated heterogeneous population of endothelial cells in HCC

**Fig. 1:**
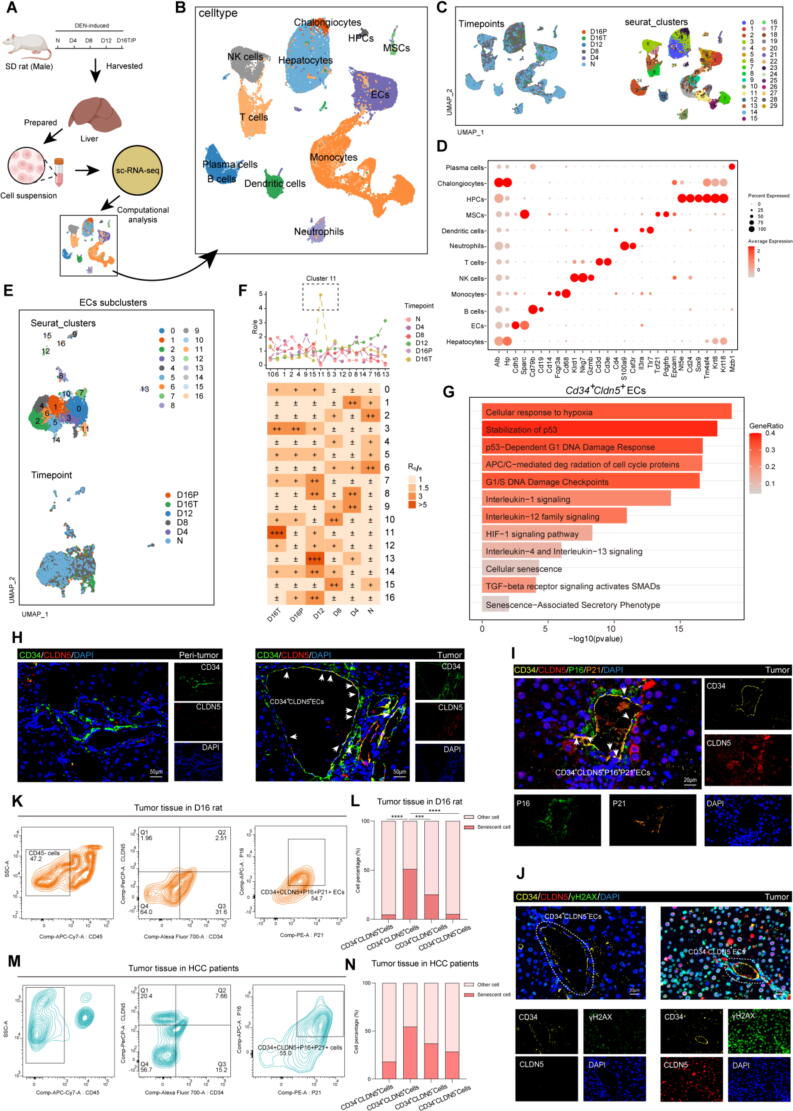
**Tumor associated senescent CD34^+^CLDN5^+^ endothelial cells existed in HCC tumor tissue.** (**A**) Schematic of the schedule for collection and processing of liver tissue for single-cell RNA sequencing. N, normal rat liver tissue; D4, liver tissue from D4 rat; D8, liver tissue from D8 rat; D12, liver tissue from D12 rat; D16T, tumor tissue from D16 rat; D16P, *peri*-tumor tissue from D16 rat. (**B-C**) UMAP plot showing the clustering results, single cells from livers exposed to DEN for different times and all cell types: Chalongiocytes, HPCs, Natural killer cells, T cells, B cells, Plasma cells, DCs, Neutrophils, MSCs, ECs, Monocytes and Hepatocytes. The different time points and cell types are indicated by different colors. (**D**) The process of categorizing and distinguishing cells based on their distinct marker gene expressions. (**E)** UMAP plot showing the ECs clustering results. Different treatment times or ECs subclusters are indicated by different colors. (**F)** The Ro/e method was used to estimate the tissue preference of each major ECs subcluster. Highlighting the Cluster 11. (**G**) Bar graphs showing the top 12 ontology terms of CD34^+^CLDN5^+^ ECs which identified by KEGG and GO enrichment analysis. (**H**) mIHC of CD34, CLDN5 in liver specimens from the D16T and D16P. Scale bars, 50 μm. (**I-J**) mIHC of CD34, CLDN5, P16, P21 and γH2AX in tumor from the D16T. Scale bars, 20 μm. (**K)** D16 rats' tumor tissue: CD34^+^CLDN5^+^ cells proportion in non-parenchymal cells, and the P16^+^P21^+^cells proportion in CD34^+^CLDN5^+^ cells were measured by flow cytometry. (**L**) Quantified data of senescent cell proportion in different non-parenchymal cells of D16 rats' tumor tissue. n = 3. Data are represented as mean. ***p < 0.001, ****p < 0.0001. (**M**) Liver cancer patients' tumor tissue: CD34^+^CLDN5^+^ cells proportion in non-parenchymal cells and the P16^+^P21^+^cells proportion in CD34^+^CLDN5^+^ cells were measured by flow cytometry. (**N**) Quantified data of senescent cell proportion in different non-parenchymal cells of HCC patients' tumor tissue. n = 2. Data are represented as mean.

The DEN-induced rat model is a well-established primary liver cancer model. The process of liver cancer development in the DEN model closely resembles that of human liver cancer. Additionally, consistent with previous reports[19], [21], we observed that the DEN-induced rat model exhibited CCA in HCC tumor tissue (Fig. S1A). Consequently, the DEN-induced rat model serves as a suitable tool for the research of the CCA formation. Our previous studies have already evaluated the liver injury status of 4 weeks (D4), 8 weeks (D8), 12 weeks (D12), 16 weeks (D16) DEN-pretreated rats. DEN-induced rats develop HCC at D12 to D16. By the end of D16, most of the rats in the study exhibited visible tumors (>1mm) in livers[22]. To obtain a single-cell atlas during the HCC, the primary liver cancer was induced in rats using 0.1 % DEN. Then, the D4, D8 and D12 rats' liver tissues, D16 rats' liver *peri*-tumor tissues (D16P) and D16 rats' liver tumor tissues (D16T) were harvested to perform single cell RNA-sequencing (10 × genomics) to compared with normal rats (Fig. 1A). After digestion and filtering (Fig. S1B and Fig. S1C), total of 37,930 cells were retained for sequencing, following gene expression normalization. Then, we performed dimensionality reduction and clustering using principal component analysis and a UMAP, respectively (Fig. 1C). According to expression of marker genes, 12 major distinct cell types were identified including chalongiocytes, hepatic progenitor cells (HPCs), natural killer cells, T cells, B cells, plasma cells, dendritic cells (DCs), neutrophils, MSCs, ECs, monocytes and hepatocytes (Fig. 1B-D). The proportion and average number of the distinct cell types in different liver samples were shown in Fig. S1D. Meanwhile, recent study has revealed a mass of the ECs (marked by CD31) are located in the tumor tissue in DEN-induced primary liver cancer model, which closely related with progression of HCC[23]. Therefore, we hypothesize that the ECs play a special role during HCC.

The ECs from 6 samples were clustered into 16 subgroups according to distinct ECs' transcriptomic signatures (Fig. 1E). Then, calculated the ratio of observed to expected cell numbers (Ro/e) using data of liver samples to quantify relative tissue enrichment of major cell clusters (Fig. 1F). The findings revealed that a distinct subgroup of ECs, denoted as cluster 11, was significantly enriched at D16T. In addition, Cluster 11 ECs showed high level of *Vim*, *Plvap*, *S100a11*, *S100a10*, *Cldn5* and *Cdkn2a*, which closely associated with cellular senescence (Fig. S1E). UMAP plots showed the Cluster 11 ECs exhibited a unique characteristic of high level of *Cd34*, *Cldn5*, *Ckdn2a* and *Tp53* (Fig. S1F). After functional enrichment analysis, the Cluster 11 ECs (CD34^+^CLDN5^+^ ECs) were found to be enriched many ontology terms which relevant to *Cellular Senescence* (Fig. 1G). As a type of cellular surface antigen, the hematopoietic progenitor cell antigen CD34, encoded by the *Cd34* gene, is expressed in endothelial progenitor cells[24]. *Cldn5* encodes claudin-5 in ECs and regulates the medium transportation in the membrane[25]. *Cdkn2a* (*p16*) encodes cyclin-dependent kinase inhibitor 2A which is one of the most prominent markers of cellular senescence (the others including CDKN1A) and plays a crucial role in cell cycle[26], [27]. *Tp53* encodes TP53-binding protein which is a master transcription and participates a great diversity of cellular responses. However, mutant *Tp53*, as a tumor-associated driver gene, is considered to trigger the release of senescence-associated secretory phenotype (SASP) factors[28], [29]. To verify the existence and characterize CD34^+^CLDN5^+^ cells, liver tissues from D16 rats were collected and multiplex immunohistochemistry (mIHC) was performed. The results showed the presence of CD34^+^CLDN5^+^ positive cells in tumor tissue, while these cells were scarcely observed in *peri*-tumor tissue (Fig. 1H). In addition, P16, CDKN1A (P21) and γH2AX were added as markers of cellular senescence and colocalized by mIHC. The results found only CD34^+^CLDN5^+^ cells were observed to be P16^+^P21^+^ and γH2AX^+^ positive, which prompted that the CD34^+^CLDN5^+^ ECs were senescent phenotype in tumor tissue (Fig. 1I-J). These results were also validated in flow cytometry (anti panel: CD45, CD34, CLDN5, P16 and P21) of D16T (Fig. 1K-L and Fig. S1G). Flow cytometry analysis showed that the CD34^+^CLDN5^+^ cells (2.51 %) existed in D16T, with more than half of these cells exhibiting senescence (P16^+^P21^+^cells, 54.7 %), which was greater than the other groups of non-parenchymal cells. Similarly, the CD34^+^CLDN5^+^ cells (7.66 %) also existed in tumor tissue of HCC patients, and were hardly detected in the *peri*-tumor tissue of HCC patients. In tumor, more than half of CD34^+^CLDN5^+^ cells were senescent cell (55.0 %) (Fig. 1M-N and Fig. S1H-I). Furthermore, primary CD34^+^CLDN5^+^ ECs were isolated from D16T by fluorescence-activated cell sorting (FACS) for culture. After characterizing the cells (Fig. S2A), most of CD34^+^CLDN5^+^ECs were P16 or P21 positive through cellular immunofluorescence staining assays (Fig. S2B). Together, these results identified that the CD34^+^CLDN5^+^ cells (cluster 11) as a subtype of ECs during the progression of HCC, which significantly existed in tumor tissue and closely related with senescence.

Tumor associated senescent endothelial cells increased cholangiocellular phenotype in HCC and recruited mesenchymal stem cells into the tumor microenvironment.

**Fig. 2:**
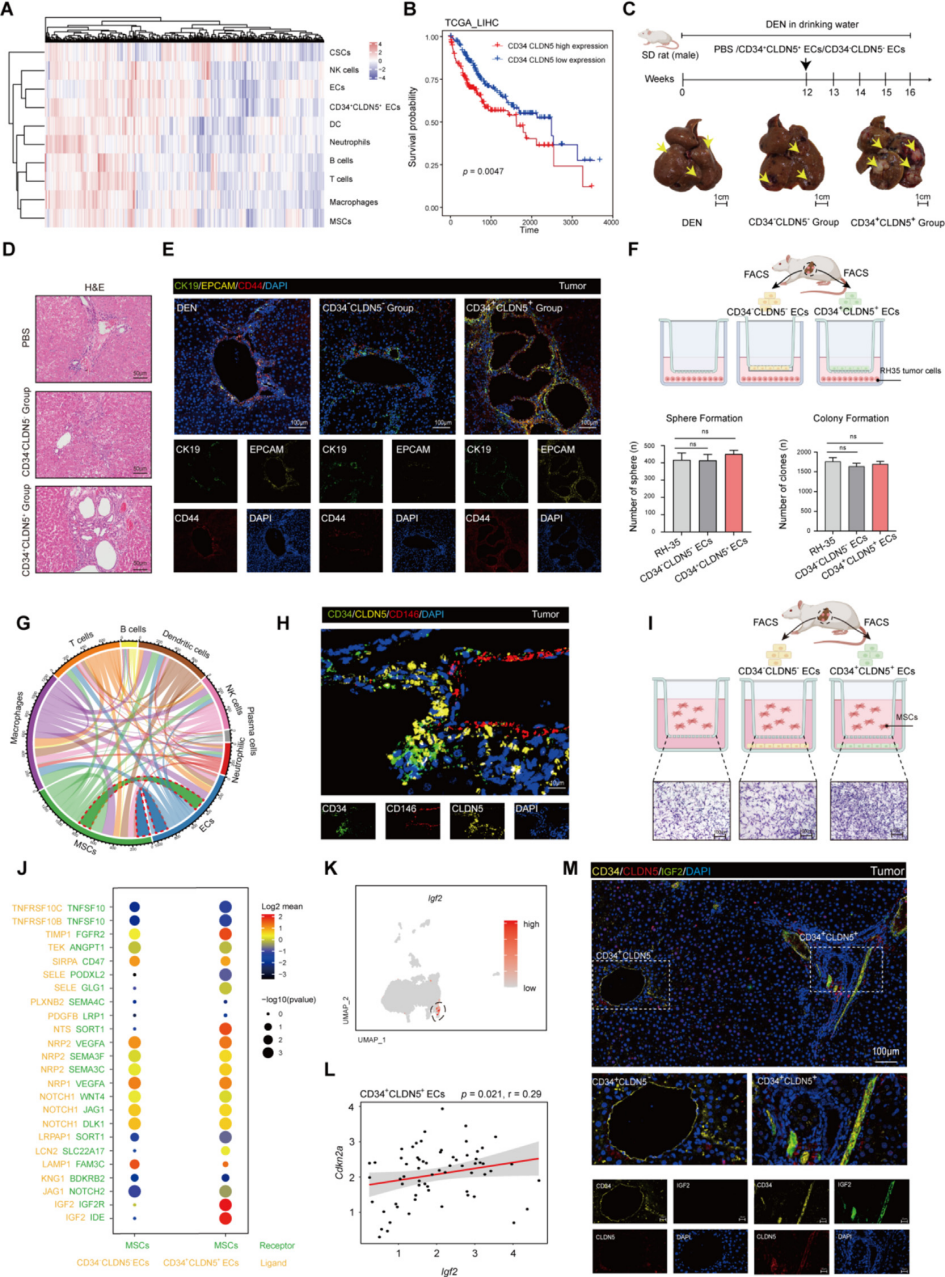
CD34^+^CLDN5^+^ endothelial cells increased the cholangiocellular phenotype within HCC and were instrumental in the recruitment of mesenchymal stem cells into the tumor microenvironment. (A) Hierarchical clustering of cell abundance predicted per sample from TCGA of HCC cohort by ssGSEA. Shown are row z-score. (B) The Kaplan-Meier overall survival curves of patients in TCGA_LIHC by the gene signature expression of CD34^+^CLDN5^+^ cells. *p* value was determined by Kaplan-Meier survival curves and log-rank test. (C) Schematic diagram for administration of CD34^+^CLDN5^+^ ECs to DEN-induced HCC rats and the gross appearance of livers exposed to DEN for 16 weeks. PBS or CD34^+^CLDN5^+^ECs/CD34^−^CLDN5^−^ECs was administered by splenic injection. CD34^+^CLDN5^+^ECs, the rats were administered CD34^+^CLDN5^+^ECs; CD34^−^CLDN5^−^ECs, the rats were administered CD34^,−^CLDN5^,−^ECs. n = 6, respectively. (D) H&E of the different tumor from the rats liver. Scale bars, 50 μm. (E) mIHC of CK19, CD44, EPCAM in different tumors. Scale bars, 100 μm. (F) Schematic diagram illustrating the co-culture setup of RH-35 cells with the ECs and Quantified data of colony and sphere formation assay of the different RH35. Data are represented as mean ± SD. ns, not significant. FACS, fluorescence-activated cell sorting. (G) Chord diagrams showing cell–cell interactions among ECs and various cell clusters in TME according to L-R pairs analysis. Circling the interaction between ECs and MSCs (H) mIHC of CD34, CLDN5, CD146 in tumor from the D16T. Scale bars, 10 μm. (I) Schematic diagram illustrating the transwell migration assay between MSCs with the ECs for 48 h. Scale bars, 100 μm. (J) Bubble chart showing the L-R pairs enrichment analysis between the MSCs and different ECs. (K) The UMAP plot showing expression levels of *Igf2* in ECs subtypes*,* with the lowest expression levels represented as gray dots and the highest expression levels as red dots. (L) The correlation (Pearson) between *Igf2* and *Cdkn2a* in CD34^+^CLDN5^+^ ECs. (M) mIHC of CD34, CLDN5 and IGF2 in tumor from the D16T. Scale bars, 100 μm. (For interpretation of the references to colour in this figure legend, the reader is referred to the web version of this article.)

In order to compare the correlation of CD34^+^CLDN5^+^ ECs with HCC, we used the single-sample gene set enrichment analysis approach to deconvolve the relative abundance of each cell type based on expression profiling data retrieved from the The Cancer Genome Atlas (TCGA) database. The scores are based on analysis of transcriptomic markers-that is, transcriptomic features that are strongly, specifically and stably expressed in a unique cell population (Fig. 2A). The result predicted that the CD34^+^CLDN5^+^ ECs closely correlated with HCC. Similarly, the higher level of CD34^+^CLDN5^+^ ECs in HCC patients was correlated with poorer survival duration according to the TCGA-LIHC database (Fig. 2B). In order to investigate the function of CD34^+^CLDN5^+^ ECs in TME, the FACS was performed to sort the CD34^+^CLDN5^+^ ECs from D16T. Then, the CD34^+^CLDN5^+^ ECs/CD34^−^CLDN5^−^ ECs/PBS were administrated by splenic injection to the D12 rats. Compared with the PBS and CD34^−^CLDN5^−^ ECs, CD34^+^CLDN5^+^ ECs developed a higher burden of liver tumors around 4 weeks after injection (Fig. 2C and Fig. S2G-H). Subsequent histological examination (H&E) and mIHC of tumor lesions revealed that CD34^+^CLDN5^+^ ECs significantly contributed to the development of CCA within HCC (Fig. 2D-E). Emerging researches have uncovered that the cancer stem-like cells (CSCs) were a major factor contributing to formation of CCA, increase in malignancy[30], [31], [32]. To investigate the underlying mechanisms by which senescent endothelial cells promote the CCA in primary liver cancer, RH-35 rat hepatocellular carcinoma cells were used to co-culture with the CD34^+^CLDN5^+^ ECs for 6 days. According to the results in vitro, the senescent ECs did not induce stem-like transformation or enhance proliferative capacity of RH-35 (Fig. 2F and Fig. S2C-F). Therefore, CD34^+^CLDN5^+^ ECs may indirectly promote the CCA through interactions with other cells in TME. Based on analysis of the ligand-receptor (L-R) pairs, the cell-to-cell interaction network in TME is shown in Fig. 2G. Interestingly, we noticed more L-R pairs were enriched in MSCs and ECs, indicating a strong intercellular communication between them. Meanwhile, mIHC showed that the presence of MSCs, identified by the markers CD146, CD90, CD73, and PDGFRβ, within TME and in proximity to the CD34^+^CLDN5^+^ ECs (Fig. 2H and Fig. S2I). Therefore, we hypothesize that the CD34^+^CLDN5^+^ ECs may recruit the MSCs into the TME. Next, the primary MSCs in rats were used to co-culture with the senescent ECs for 2 days. The co-culture assays indicated that CD34^+^CLDN5^+^ ECs significantly recruited the MSCs and enhanced their migratory ability (Fig. 2I and Fig. S2J-L). Additionally, a set of IGF2-IGF2R related L-R pairs was significantly enriched in the MSCs and CD34^+^CLDN5^+^ ECs (Fig. 2J). UMAP plots showed that the CD34^+^CLDN5^+^ ECs exhibited the high level of *Igf2* (Fig. 2K). Correlation analyses provided a positive correlation between *Igf2* and *Cdkn2a* in CD34^+^CLDN5^+^ ECs (Fig. 2L). As a member of SASP, insulin-like growth factor 2 (IGF2) and its binding protein, mainly produced by the liver for regulating organ development, growth and regeneration. It was reported that IGF2 enhanced the migration and growth potential of tumor-derived cells in vitro and in vivo. After the resection of primary tumors, the cancer cells which expressed the IGF2 binding protein 1 (IGF2BP1) were observed metastasize. In addition, the deletion of IGF2BP1 in HCC-derived HepG2 cells inhibited the tumor growth during the Xenograft studies[33], [34], [35]. To explore the potential mechanisms underlying the recruitment of MSCs by senescent ECs, we performed mIHC in tumor. The results demonstrated that the IGF2 mainly expressed at CD34^+^CLDN5^+^ double positive cells in tumor tissue (Fig. 2M). Therefore, the collective data suggest CD34^+^CLDN5^+^ ECs promoted the development of CCA and recruited MSCs into TME, with these ECs exhibiting elevated expression of IGF2 within the tumor.

Mesenchymal stem cells presented a tumor-promoting effect in HCC through promoting the cholangiocellular phenotype formation

**Fig. 3:**
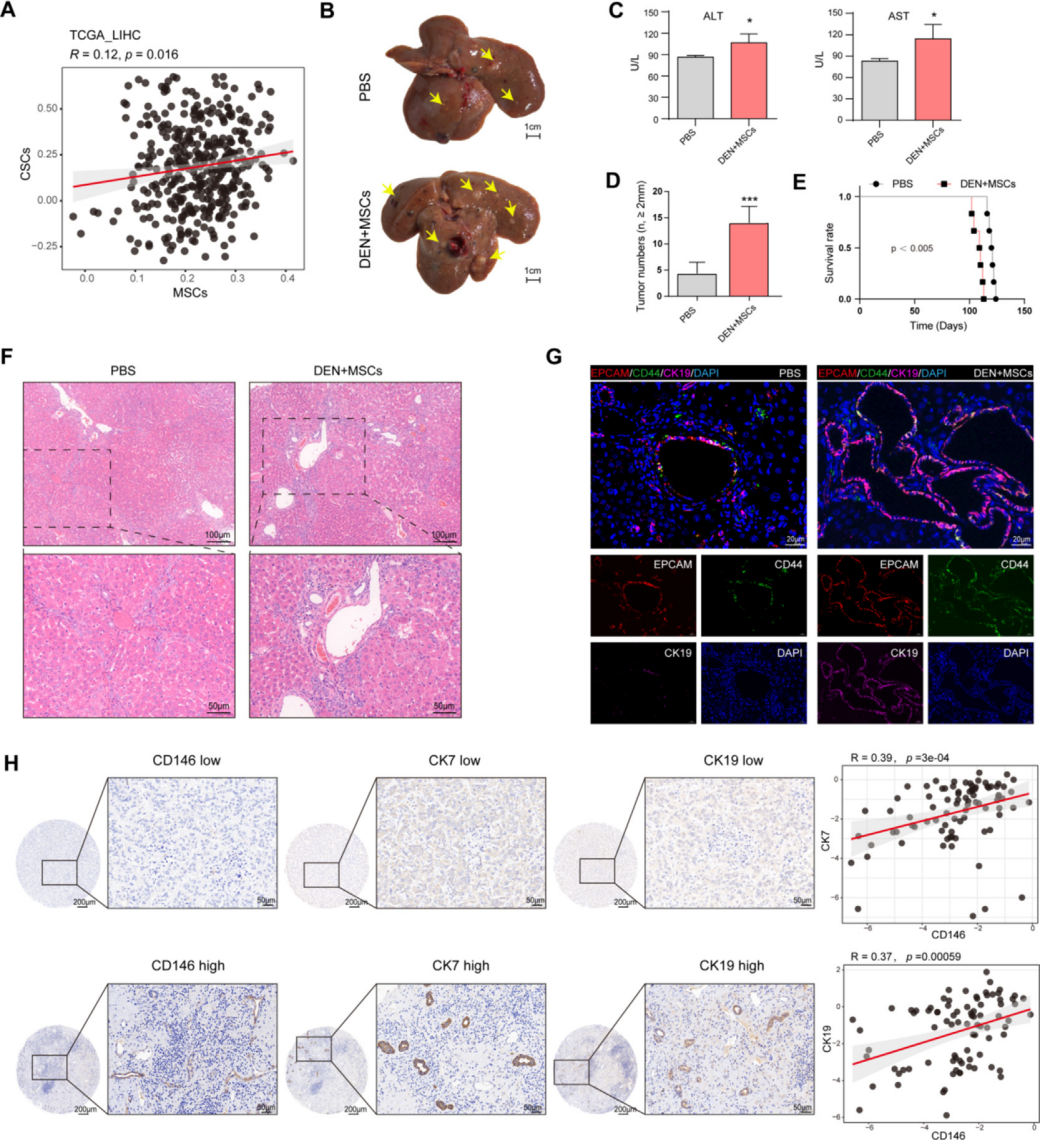
**Mesenchymal stem cells had tumor-promoting effect through promote the cholangiocellular phenotype formation.** (**A)** The correlation analysis between MSCs and CSCs according to TCGA_LIHC. (**B)** The gross appearance of livers of rats exposed to DEN for 16 weeks. PBS, the liver was from PBS administrated DEN-induced rats; DEN + MSCs, the liver was from MSCs administrated DEN-induced rats. The yellow arrows show tumor nodules. (**C)** Serum levels of AST and ALT were determined to indicate the extent of liver damage. Data are represented as mean ± SD. *p < 0.05. (**D)** The number of tumor nodules (Diameter ≥ 2 mm) per liver in D16 rats. Data are represented as mean ± SD. ***p < 0.001. n = 6, respectively. (**E)** The Kaplan-Meier overall survival curves of rats exposed to DEN. *p* value was determined by Kaplan-Meier survival curves and log-rank test. n = 6, respectively. (**F)** H&E of liver specimens from the D16T of DEN + MSCs group and PBS group. Scale bars, 100 μm and 50 μm. (**G)** mIHC of CK19, CD44, EPCAM in liver specimens from the D16T of DEN + MSCs group and PBS group. Scale bars, 20 μm. **(H)** Left six panels: CD146, CK7 and CK19 expression was detected in tumor tissues from 66 patients with liver cancer by IHC. Scale bars, 200 μm and 50 μm. Right two panels: the correlation analysis between CK7 and CD146, CK19 and CD146. (For interpretation of the references to colour in this figure legend, the reader is referred to the web version of this article.)

According to the TCGA-LIHC database, a positive correlation between MSCs and CSCs was obtained (Fig. 3A). In order to explore the effect of MSCs on HCC, the MSCs and PBS were administrated by tail vein to the D12 rats once a week. Compared with the PBS, injection of MSCs significantly increased the tumor burden, exacerbated the progression of HCC and abbreviated the survival time of the rats (Fig. 3B-E). H&E of tumor lesions revealed that CCA was more severe in DEN + MSCs group compared to the PBS (Fig. 3F). In the mIHC, the CCA, marked by CK19, EPCAM and CD44 was observed, further indicating an increase in the CCA and heightened tumor malignancy in DEN + MSCs group (Fig. 3G). The similar results were shown in patients. The positive correlations were also obtained among the CK7, CK19 and CD146 expression levels according to IHC from 66 primary liver cancer patients, which suggested that the MSCs were increasing along with the increase of CCA within HCC (Fig. 3H). All data demonstrated that the MSCs increased the CCA to exacerbate the progression of HCC.

### Mesenchymal stem cells enhanced the stemness features of HCC cells by secretome

**Fig. 4:**
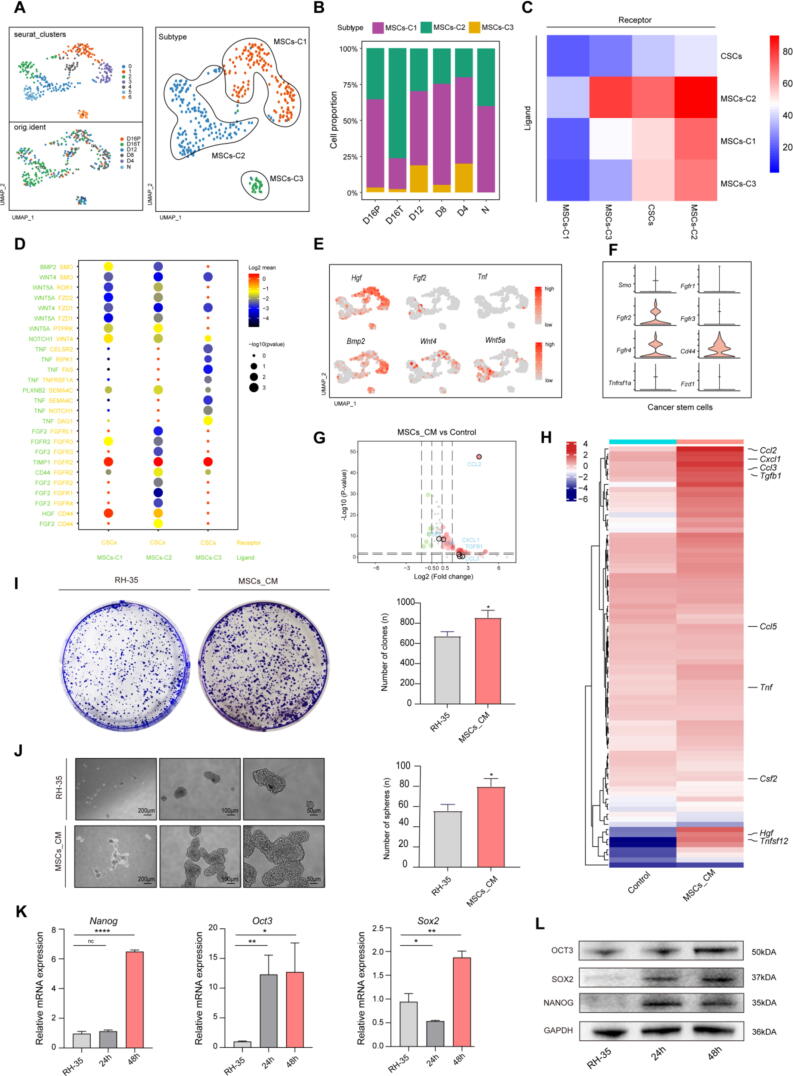
Mesenchymal stem cells enhanced the cancer stem cell-like features of HCC cells via secreting the inflammatory and trophic Factors. (A) UMAP plot showing the MSCs clustering results. The different time points and MSCs subtypes are indicated by different colors. (B) The proportion of the different MSCs subtypes in different liver samples. (C) Heatmap of L-R enrichment analysis between CSCs and different MSCs subtypes, with the fewest L-R pairs number represented as blue boxes and the most L-R pairs number as red boxes. (D) Bubble chart showing the L-R pairs enrichment analysis between the CSCs and different MSCs subtypes. (E) The UMAP plot showing expression levels of *Hgf, Bmp2, Wnt4 and Wnt5a*. (F) Violin plots showing the expression levels of selected genes in cancer stem cells' receptors. (G) Volcano plots showing the significant differences in protein cargo between the normal DMEM medium and MSCs conditional medium. Highlighting the inflammatory and trophic associated protein. (H) The heat map of differences in the protein cargo between the normal DMEM medium and MSCs conditional medium. (I) Clonogenic ability of RH-35 cells upon the different medium exposure was examined using colony formation assay. A representative photograph is provided in the left panel, and quantified data are shown in the right panel. RH-35, RH-35 exposed in normal DMEM medium; MSCs_CM, RH-35 exposed in MSCs conditional medium. Data are represented as mean ± SD. *p < 0.05. (J) Sphere ability of RH-35 cells upon the different medium exposure was examined using sphere assay. A representative photograph is provided in the left panel, and quantified data are shown in the right panel. Data are represented as mean ± SD. *p < 0.05. (K) Expression of stemness-related mRNA which folded over *Gapdh* was examined by RT-PCR in RH-35 treated with MSC_CM for 0 h, 24 h and 48 h. Data are represented as mean ± SD. **p < 0.01, ***p < 0.001, ****p < 0.0001. L Expression of stemness-related protein was examined by WB in RH-35 treated with MSC_CM for 0 h, 24 h and 48 h. (For interpretation of the references to colour in this figure legend, the reader is referred to the web version of this article.)

Recent studies noticed MSCs promote cancer cell proliferation and induce breast cancer cells to become CSCs through cytokine networks, leading to cancer progression[36], [37]. Thus, we hypothesize that MSCs may induce stem-like transformation in tumor cells, resulting in an increased CCA. In order to gain insights into the potential regulatory relationships between the CSCs and MSCs, the MSCs were focused and then were clustered into 3 subgroups with distinct sets of marker genes (Fig. 4A and Fig. S3A), named as MSCs-C1, MSCs-C2 and MSCs-C3. After functional enrichment analysis, MSCs were observed to have high relevant ontology terms about *Epithelial to mesenchymal transition*, *Angiogenesis*, *Protein secretion*, *WNT-beta-catenin signaling* and *TNF-alpha signaling* (Fig. S3B). The proportion and the number of the different subtypes in different liver samples were shown in Fig. 4B and Fig. S3C. The heat map of L-R pairs enrichment analysis was shown in Fig. 4C, implying that the CSCs interacted closely with the MSCs. Moreover, multiple L-R pairs related to TNF, WNT and FGF-related signaling pathways were found to be enriched in MSC subtypes and CSCs. These pairs were closely associated with the CSCs activation, maintenance of stemness, and proliferation. Additionally, UMAP plots revealed that ligands of *Hgf*, *Bmp2*, *Wnt4*, *Tnf*, *Fgf* and *Wnt5a* were highly expressed in MSCs, which corresponded with the high expression of receptors *Cd44*, *Fgfr2*, and *Fgfr4* in CSCs (Fig. 4E-F). Thus, we hypothesise that the MSCs secrete the inflammtory and proliferative associated factors to maintain the CSCs features. To investigate the mechanism underlying the cross-talk between MSCs and CSCs, the MSCs conditional medium (MSC-CM) was collected and performed to Olink Proximity Extension Assay. The heat map of differences in the protein cargo between the MSCs-CM and complete medium was shown in Fig. 4H, highlighting the presence of inflammatory and trophic-related proteins, which were enriched in MSCs-CM. Volcano plots indicated that CCL2, CCL5, TNF, TGF-β, and GM-CSF were up-regulated in MSC-CM (Fig. 4G). Then, the MSC-CM was used to culture the hepatoma carcinoma cell line. According to the in vitro results, the MSC-CM significantly induced stem-like transformation and enhanced proliferative capacity in RH-35 (Fig. 4I-L). All the results illustrated the MSCs had robust communication with CSCs, secreting a spectrum of inflammatory and trophic factors that contribute to the induction of stem-like transformation in HCC cells.

### Mesenchymal stem cells were recruited to the TME through IGF2/IGF2R signaling

**Fig. 5:**
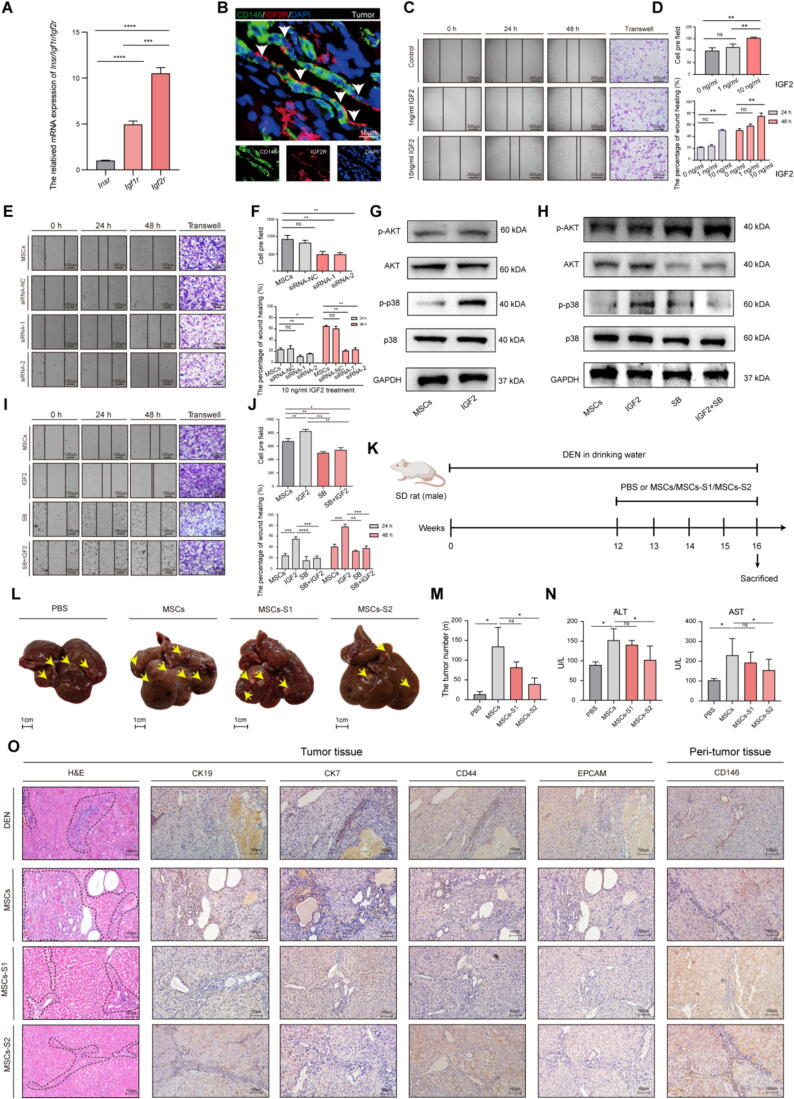
**The endothelial cells recruited the mesenchymal stem cells into the tumor microenvironment via IGF2/IGF2R signaling.** (**A)** Expression of *Igf2r*, *Insr* and *Igf1r* which folded over *Gapdh* was examined by RT-PCR in primary MSCs. Data are represented as mean ± SD. ***p < 0.001, ****p < 0.0001. **(B)** mIHC of CD146 and IGF2R in tumor from the D16T. Scale bars, 15 μm. (**C)** Transwell migration assay for rat primary MSCs treated with IGF2 (0 ng/ml, 1 ng/ml and 10 ng/ml) for 48 h. Wound healing assay for rat primary MSCs treated with the same way for 0 h, 24 h and 48 h. The black main strings show the wound area. Scale bars, 200 μm. (**D)** Quantified data of transwell migration assay and wound healing assay of primary MSCs treated with IGF2. Data are represented as mean ± SD. **p < 0.01; ns, not significant. **(E)** Transwell migration assay for rat primary MSCs treated with *Igf2*r-siRNA (100 nM) or scramble siRNA (as negative control) or not (as Control) for 48 h. Wound healing assay for rat primary MSCs treated with the same way for 0 h, 24 h and 48 h. SiRNA-1, *Igf2*r-siRNA candidate 1; SiRAN-2, *Igf2*r-siRNA candidate 2; SiRAN-NC, negative control. Scale bars, 100 μm. (**F)** Quantified data of transwell migration assay and wound healing assay of MSCs treated with *Igf2*r-siRNA. Data are represented as mean ± SD. *p < 0.05. (**G)** Expression of MAPK and PI3K signaling pathway related protein was examined by WB assay in rat primary MSCs treated with 10 ng/ml IGF2 or not for 48 h. (H**)** Expression of MAPK and PI3K signaling pathway related protein was examined by WB in rat primary MSCs treated with 10 ng/ml IGF2 and/or p38 MAPK signaling inhibitor (as SB) or not for 48 h. **(I)** Transwell migration assay for rat primary MSCs treated with 10 ng/ml IGF2 and/or p38 MAPK signaling inhibitor (SB) or not for 48 h. Wound healing assay for rat primary MSCs treated with the same way for 0 h, 24 h and 48 h. **(J)** Quantified data of transwell migration assay and wound healing assay of MSCs treated with IGF2 and/or SB. Data are represented as mean ± SD. (**K)** Schematic diagram for administration of MSCs to DEN-induced HCC rats. PBS or MSCs/MSCs-S1/MSCs-S2 (n = 3, respectively) was administered by intravenous injection. MSCs-S1, IGF2R silent MSCs by *Igf2*r-siRNA candidate 1; MSCs-S2, IGF2R silent MSCs by *Igf2*r-siRNA candidate 2. (L**)** The gross appearance of livers of rats exposed to DEN for 16 weeks. The yellow arrows show tumor nodules. PBS, the rats were administrated by PBS; MSCs, the rats were administrated by MSCs; MSCs-S1, the rats were administrated by MSCs which treated with *Igf2*r-siRNA candidate 1; MSCs-S2, the rats were administrated by MSCs which treated with *Igf2*r-siRNA candidate 2. (**M)** The number of tumor nodules per liver in different D16 rats. Data are represented as mean ± SD. **(N)** Serum levels of AST and ALT were determined to indicate the extent of liver damage. Data are represented as mean ± SD. **(O)** Left three panels: H&E of liver specimens from the different D16T. Scale bars, 100 μm. Dotted portion shows the CCA area. Right fifteen panels: IHC with antibodies CK19, CK7, CD146, EPCAM and CD44 for the different liver specimens from the D16T and D16P. Scale bars, 100 μm. (For interpretation of the references to colour in this figure legend, the reader is referred to the web version of this article.)

The locally generated IGF2 at tumor sites contributes to postnatal vasculogenesis by augmenting the recruitment of bone marrow-derived endothelial progenitor cells via IGF2-IGF2R system[38]. To determine whether the MSCs were recruited to the TME via the similar mechanism, the primary MSCs were isolated from rat to perform the RT-PCR. The relative mRNA level of *Igf2r* was significantly higher than that of other IGF2 binding receptors including *Igf1r* and *Insr* (Fig. 5A). Similarly, the results of mIHC of D16T sample demonstrated the co-localization of CD146 and IGF2R, as presented in Fig. 5B, indicating the high expression of IGF2R in MSCs. The effects of IGF2 on MSCs' migratory capacity were evaluated using both wound healing and transwell assays. Our observations indicated that the medium supplemented with IGF2 at concentrations ranging from 0 to 10 ng/ml, particularly at a concentration of 10 ng/ml, significantly enhanced the recruitment of MSCs and markedly improved their migration capacity (Fig. 5C-D). In order to substantiate our observations regarding the contribution of IGF2-IGF2R system to recruiting MSCs, the expression of *Igf2r* mRNA and IGF2R in MSCs was silenced by siRNA (Fig. S3D-E). According to the results in vitro, silencing of the IGF2R significantly inhibited the migratory capacity of MSCs (Fig. 5E-F). It is known that IGF2 activates downstream PI3K and MAPK pathways, which regulate cell proliferation, invasion, migration, and angiogenesis[39], [40], [41]. To determine which downstream signaling pathways were responsible for enhancing the MSCs' migration capacity, the expressions of phosphorylated(p)-p38 MAPK/p-38 MAPK and p-AKT/AKT were detected in MSCs which were cultured by 10 ng/ml IGF2 medium or non-IGF2 medium. Although the results indicated no significant differences in AKT, p-AKT, and p38 MAPK expression between the IGF2-treated group and the control group, the expression of p-p38 MAPK in the IGF2 group was noticeably higher than in the control group (Fig. 5G). We surmised that IGF2-IGF2R may activate the MAPK signaling in MSCs. To test this hypothesis, the p-p38 MAPK inhibitor was utilized to inhibit the MAPK signaling. The results demonstrated that MSCs treated with a combination of IGF2 and p-p38 MAPK inhibitor exhibited decreased levels of both p-p38 MAPK and AKT compared to other treatment groups. However, an increase in p-AKT was observed, suggesting that the MAPK signaling pathway was blocked by the p-p38 MAPK inhibitor (Fig. 5H). Moreover, after suppressing the MAPK pathway, the migration ability of MSCs significantly decreased and few of MSCs crossed the micropore into the IGF2 medium (Fig. 5I-J).

Based on the potential effect of IGF2/IGF2R axis, we asked whether the MSCs were recruited to the TME by the IGF2 (secreted by CD34^+^CLDN5^+^ cells) and contributed to increase the CCA within HCC. In the D12 rats, MSCs with silenced IGF2R, MSCs, and PBS were administered once a weeks till the age of D16 (Fig. 5K). Although the rats in the silenced IGF2R-MSCs group showed an aggravated the progression of HCC which compared to PBS group, they exhibited alleviation in the liver injury, tumor burden and the number of dysplastic nodules compared to the MSCs group (Fig. 5L-N). H&E showed that the silent IGF2R*-*MSCs group exhibited decreased foramtion of CCA in comparison to the MSCs group (Fig. 5O). In the IHC staining of tumor tissue, the CCA-positive area, marked by CK19, CK7, EPCAM, and CD44, in silent IGF2R*-*MSCs groups were lower than those in the MSCs group (Fig. 5O). Additionally, compared to the MSCs group, a few MSCs were detected near the CD34^+^CLDN5^+^ cells in the HCC tissues of silent IGF2R*-*MSCs group rats. Conversely, the gene knockdown MSCs were predominantly localized in the peritumoral tissue. (Fig. 5O and Fig. S3F). All the data revealed that the CD34^+^CLDN5^+^ cells secreted the IGF2 to recruit the MSCs into the TME via IGF2/IGF2R/MAPK signaling, leading to CCA formation.

### IGF2 expression in tumor associated senescent endothelial cells was regulated by transcription factor CEBPβ

**Fig. 6:**
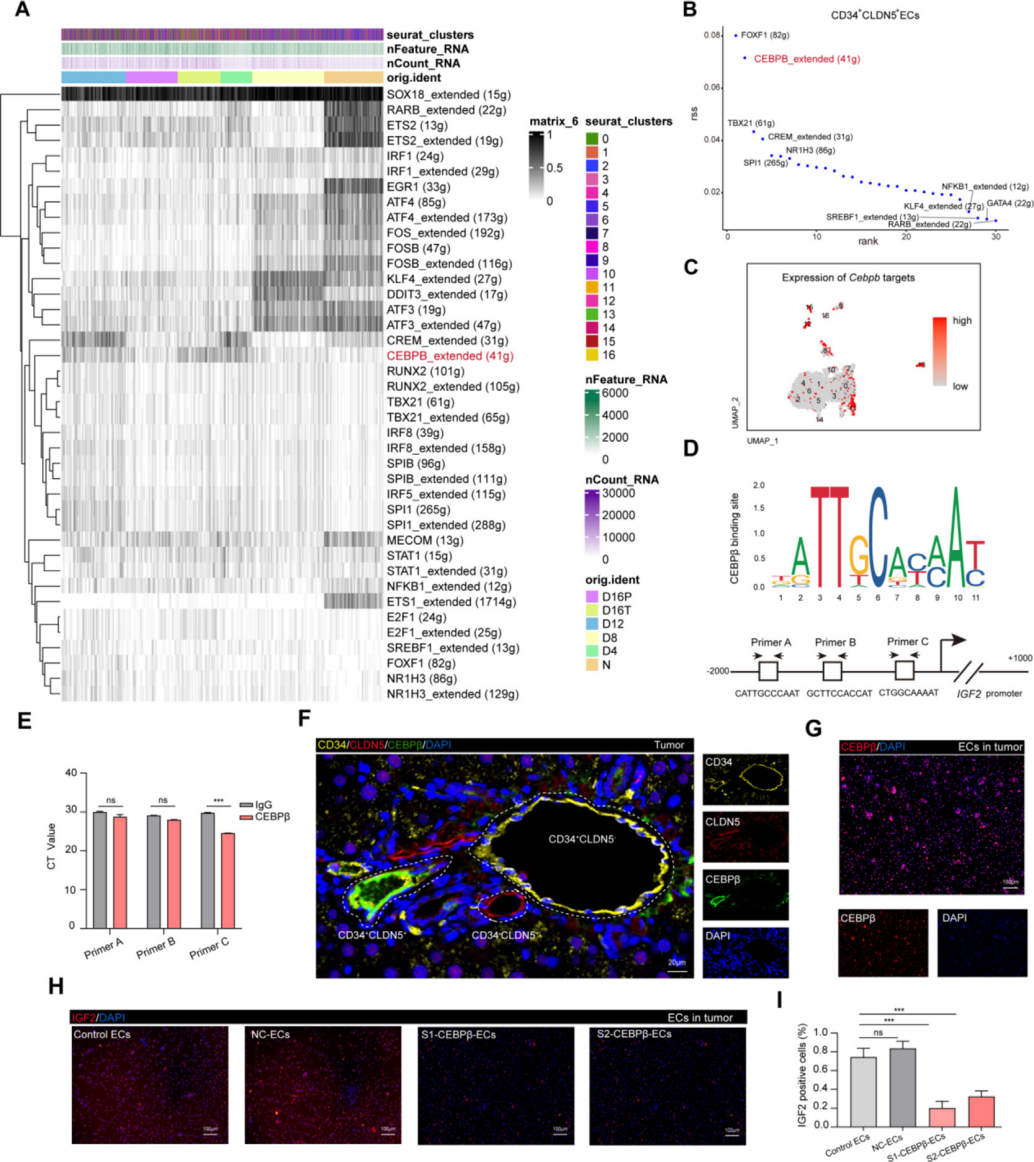
**CEBPβ bound with *Igf2*-promoter-sequence in hepatic senescent endothelial cells to up regulate the IGF2 expression. (A)** Heatmap of the AUC scores of expression regulation by transcription factors, as estimated using SCENIC, for each of the ECs from 16 clusters. Shown are the transcription factors having the highest difference in expression regulation estimates among different times ECs from livers of rats exposed to DEN. (**B)** Shown the Top 30 transcription factors having the significant difference in expression regulation in CD34^+^CLDN5^+^ ECs. (**C)** The UMAP plot showing expression levels of *Cebpb* targets in ECs subtypes. (**D)** The Schematic diagram of canonical CEBPβ-binding motif (from JASPAR Database) and three potential CEBPβ responsive elements (Primer A, Primer B, and Primer C) in the IGF2 promoter region. (**E)** Enrichment of the fragments containing the CEBPβ binding sites within the IGF2 promoter in CD34^+^CLDN5^+^ECs by CUT&Tag-qPCR. Comparing to the background DNA fragment pulled down by IgG immunoprecipitation. Data are presented as mean ± SD. ***p < 0.001. (**F)** mIHC of CD34, CLDN5 and CEBPβ in tumor from the D16T. Scale bars, 20 μm. (**G)** Immunofluorescence staining of CEBPβ in CD34 + CLDN5 + ECs from D16 rats and normal 6 month-old normal rat livers. Scale bars, 100 μm. **(H)** Immunofluorescence staining of IGF2 for CD34^+^CLDN5^+^ ECs from D16T treated with *Cebpb*-siRNA (100 nM) or scramble siRNA (as negative control) or not for 48 h. S1-CEBPβ-ECs, CD34^+^CLDN5^+^ ECs which treated with *Cebpb*-siRNA candidate 1; S2-CEBPβ-ECs, CD34^+^CLDN5^+^ ECs which treated with *Cebpb*-siRNA candidate 2; NC-ECs, negative control. Scale bars, 100 μm. (**I)** Quantified data of immunofluorescence staining of IGF2 in CD34^+^CLDN5^+^ ECs. Data are presented as mean ± SD. ns, no significant, ***p < 0.001.

The expression of SASP depends on the regulation of transcriptional factors. In order to investigate which transcriptional factors control the *Igf2* gene expression, we performed an enrichment analysis of transcriptional factors (TFs) using our scRNA-seq data (Fig. 6A). The results suggested that CEBPβ TFs were mainly enriched at D16T, and presented high level in CD34^+^CLDN5^+^ ECs subtype (Fig. 6B). UMAP plots showed that the targets of *Cebpb* exhibited high expression, particularly in CD34^+^CLDN5^+^ cells (Fig. 6C). CCAAT/enhancer-binding protein beta (CEBPβ), an inflammatory transcription factor, which directly binds to DNA and enhances/activates the expression of SASP genes such as IL1A, IL6, and IL8[42], [43], [44]. Based on the potential contribution of CEBPβ to the senescence-associated cells, we hypothesized that CEBPβ binds to the *Igf2-*promoter sequence and enhanced the expression of IGF2. The JASPAR website predicted that the CEBPβ motif can bind to three binding sites in the *Igf2* promoter (Fig. 6D). The corresponding primers were designed accordingly. Then, the CUT&Tag technique was performed to construct the DNA library of CD34^+^CLDN5^+^ ECs from the D16T samples. The CUT&Tag-qPCR and ESMA assays revealed that CEBPβ binds to one high-affinity E-box (Binding site: CTGGCAAAAT) in the *Igf2* promoter (Fig. 6E and Fig. S3G). To confirm whether the CEBPβ was expressed in CD34^+^CLDN5^+^ ECs, mIHC of rat liver tissues was performed and observed that the CEBPβ mainly expressed at CD34^+^CLDN5^+^ double positive cells in tumor tissue of D16 rat (Fig. 6F). Similarly, the primary CD34^+^CLDN5^+^ ECs from D16T sample showed the expression of CEBPβ (Fig. 6G). Furthermore, siRNA was utilized to silence the expression of CEBPβ in D16T CD34^+^CLDN5^+^ ECs (Fig. S3H), resulting in a distinct reduction of fluorescence intensity in IGF2 positive cells (Fig. 6H-I). All together, we concluded that the CEBPβ combined with the DNA sequence of *Igf2-*promoter in CD34^+^CLDN5^+^ ECs to enhance the IGF2 expression.

Tumor associated senescent endothelial cells commonly existed in intrahepatic cholangiocarcinoma and combined hepatocellular carcinoma-cholangiocarcinoma, correlating with short survival duration of liver cancer patients.

**Fig. 7:**
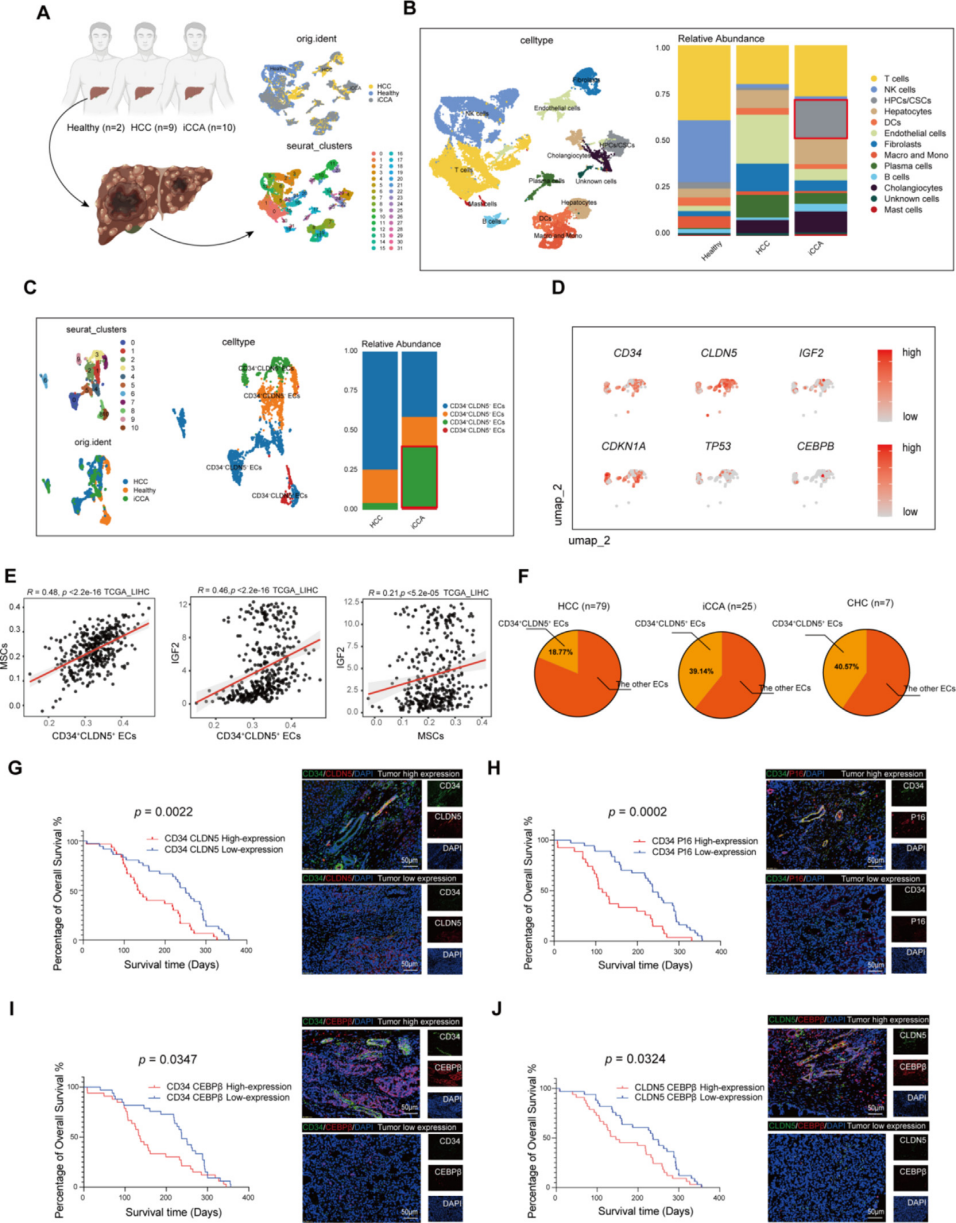
Tumor associated senescent endothelial cells are frequently observed in both intrahepatic cholangiocarcinoma and combined hepatocellular carcinoma-cholangiocarcinoma, and their presence is significantly correlated with a short survival duration in patients with liver cancer. (A-B) Schematic of processing of liver tissue for single-cell RNA sequencing, UMAP plot showing the clustering results, single cells from different liver tissue and relative abundance of the different cell types in different liver samples: Chalongiocytes, HPCs/CSCs, NK cells (Nature kill cells), T cells, B cells, Plasma cells, DCs, Neutrophils, Endothelial cells, Macro and Mono (Macrophages and Monocytes), Mast cells, Fibrolasts and Hepatocytes. The different samples and cell types are indicated by different colors. Highlight the CSCs in iCCA. Healthy, the liver tissue from the patients without HCC. HCC, the tumor from the patients with HCC. iCCA, the tumor from the patients with iCCA. (C) UMAP plot showing the ECs clustering results and relative abundance of the different ECs subtypes in HCC and iCCA. The different samples and ECs subtypes are indicated by different colors. Highlight the CD34^+^CLDN5^+^ ECs in iCCA. (D) The UMAP plot showing expression levels of *CEBPB*, *CD34*, *CLDN5*, *IGF2*, *CDKN1A* and *TP53* in CD34^+^CLDN5^+^ECs. (E) The correlation analysis between IGF2 and CD34^+^CLDN5^+^ECs, IGF2 and MSCs, CD34^+^CLDN5^+^ECs and MSCs according to TCGA_LIHC. (F) The pie chart illustrating the proportion of CD34^+^CLDN5^+^ECs relative to the total ECs across various liver cancer. (G-J) Left four panels: the Kaplan-Meier overall survival curves of tumor tissues from 66 patients with liver cancer by co-expression of CD34, CLDN5, P16 and CEBPβ in mIHC. *p* value was determined by Kaplan-Meier survival curves and log-rank test. Right eight panels: CD34, CLDN5, p16 and CEBPβ co-expression was detected in tumor tissues from 66 patients with HCC by mIHC. Scale bars, 50 μm.

To survey the expression of the tumor associated senescent ECs in different types of primary liver cancer, we consulted scRNA seq data of 21 clinic samples, including 2 from healthy livers, 9 with HCC and 10 with iCCA (Fig. 7A, Data from GSE125449 and GSE136103). A total of 14 cell clusters were identified among 35,952 cells obtained. Similar with our finding, the CSCs enriched in the iCCA (Fig. 7B and Fig. S4). Then, the ECs were further analyzed and clustered into 4 subgroups according to the *CD34* and *CLDN5* expression. In comparison to HCC, CD34^+^CLDN5^+^ ECs exhibited a higher relative abundance within iCCA (Fig. 7C). The level of *IGF2*, *CEBPB*, *TP53*, and *CDKN1A* was also high in CD34^+^CLDN5^+^ ECs (Fig. 7D). Subsequently, we asked the potential association of CD34^+^CLDN5^+^ ECs, MSCs and IGF2 in patients according to TCGA database. The positive correlations between CD34^+^CLDN5^+^ ECs and IGF2, MSCs and IGF2, CD34^+^CLDN5^+^ ECs and MSCs were obtained (Fig. 7E), implying that the IGF2 was a medium for communication between MSCs and CD34^+^CLDN5^+^ ECs. In order to enhance our results confidence, the scRNA-seq analysis of 160 samples from 124 treatment-naive patients, including 79 with HCC, 25 with iCCA and 7 with cHCC-CCA (Data from https://meta-cancer.cn:3838/scPLC) were consulted[45]. We noticed that in patients with CCA (iCCA and cHCC-CCA), the proportion of CD34^+^CLDN5^+^ ECs to the total ECs is close to half (Fig. 7F). Furthermore, to investigate the correlation of CD34^+^CLDN5^+^ ECs with overall survival duration in primary liver cancer patients, mIHC were performed on tissue microarrays containing 66 primary liver cancer specimens with long-term clinical follow-up data. Similarly, the CD34^+^CLDN5^+^ cells were existed in tumor samples of patient. The patients were divided into eight groups: CD34^High^CLDN5^High^ (30 of 66, 45.6 %), CD34^Low^CLDN5^Low^ (36 of 66, 54.5 %), CD34^High^p16^High^ (27 of 64, 42.2 %, 2 patient specimens with suboptimal staining were excluded), CD34^Low^p16^Low^ (37 of 64, 57.8 %, 2 patient specimens with suboptimal staining were excluded), CD34^High^CEBPβ^High^ (33 of 66, 50.0 %), CD34^Low^CEBPβ^Low^ (33 of 66, 50.0 %), CLDN5^High^CEBPβ^High^ (33 of 66, 50.0 %), CLDN5^Low^CEBPβ^Low^ (33 of 66, 50.0 %) according to the colocalizated area, respectively. Moreover, the patients in CD34^High^p16^High^ (Mean survival time: 143.81 days vs. 230.81 days, Log rank test, *p* = 0.0002), CD34^High^CLDN5^High^ (Mean survival time: 164.27 days vs. 212.29 days, Log rank test, *p* = 0.0022), CD34^High^CEBPβ^High^ (Mean survival time: 163.54 days vs. 225.76 days, Log rank test, *p* = 0.0347), CLDN5^High^CEBPβ^High^ (Mean survival time: 170.70 days vs. 219.63 days, Log rank test, *p* = 0.0324) groups had a lower overall survival duration than the other groups (Fig. 7G-I).

## Discussion

In primary liver cancer, the formation of CCA in HCC is closely associated with the poor prognosis and high tumor malignancy. However, the underlying mechanism of the CCA formation is not completely understood. With in-depth knowledge of HCC, the researchers have gradually noticed the significance of senescent cell for the cancer. Emerging research suggests that senescent cells secrete the SASP factors to promote tumor development and malignant phenotypes by enhancing the cancer cellular proliferation and invasiveness in TME. Heikenwalder *et al*. found that senescent cells expressed high levels of IL-6, activating the hepatic progenitor cells and causing the cHCC-CCA tumors[31]. Eggert *et al*. revealed that senescent hepatocytes secreted the CCL2 to recruit the immunosuppressive myeloid cells and promoted growth of HCC[46]. Nonetheless, the specific mechanism by which the senescent host cells that contribute to the construction of the TME remains unclear. Within our project, sc-RNA seq analysis was employed to explore the heterogeneity of ECs throughout the initiation and progression of primary liver cancer, and identified a unique subset of tumor associated senescent CD34^+^CLDN5^+^ECs, which were mainly present in the tumor tissue. In addition, the tumor associated senescent ECs promoted the CCA in our DEN model and were closely related with the survival duration of 66 liver cancer patients. Generally, ECs in the TME, especially liver sinusoidal endothelial cells, participate in angiogenesis to bring ample nourishing factors for the shared onco-sphere. In addition, the ECs express multiple cytokines to recruit other tissue derived tumorigenic cells towards the liver. Similarly, when cells undergo senescence, enhancer regions of hundreds of SASP factors are made accessible and transcribed by NF-κB, CEBPβ, and other factors to intensify the secretion of inflammatory cytokines and chemokines, which deliver the damage signaling for surrounding cell[29]. Therefore, we suggested that the senescent ECs in TME released the SASP to recruit the tumor-associated cell, so as to increase CCA.

According to our scRNA-seq data, we noticed a strong interaction relationship between ECs and MSCs. Meanwhile, we found that the tumor associated senescent ECs recruited MSCs into TME. As a type of stromal cells in TME, MSCs have multilineage potential differentiation ability and secrete cytokines to induce tissue repair. Interestingly, many documents support that MSCs exhibit strong immunomodulatory capacity and migrate into the TME through inflammation. Then, the endogenous and exogenous MSCs unite the tumor associated cells to trigger cross-talks with cancer cell to create a dynamic environment that supports the tumor progression[47], [48]. In addition, MSCs facilitate the cancer cell proliferation and induce cancer cell to CSCs via cytokines networks, resulting in the cancer deterioration[36], [37]. Ling *et al*. found that stromal derived factor-1/CXCR4 axis regulated MSCs migration to the injured Liver. Our previous study also noticed that a number of MSCs existed in liver tumor tissue and participated in the construction of the inflammatory-related TME[49], [50]. In addition, MSCs in TME promoted HCC progression by secreting various cytokines such as IL-10, TGF[51], [52]. However, lack of the specific manner to determine which type of the cell in TME that recruit the MSCs and the mechanism of the cancer promoting is not completely understood. Here, scRNA-seq data provided a suggestive conclusion of a closely interaction between MSCs and CSCs. According to Olink Proximity Extension assay and evidences in vivo and vitro, we revealed that the MSCs secreted multiple cytokines, including CCL2, CCL25, CXCL1, TGF-β and GM-CSF to enhance the CSC-like characteristics in HCC cell, and accelerated the formation of the tumor malignancy via increase the CCA. Although we mainly observed that senescent ECs increased the CCA by recruiting MSCs in our study, some results suggest that senescent endothelial cells and MSCs may also be involved in constructing the tumor immune microenvironment. Fig. 2G indicated that both MSCs and ECs have significant intercellular interactions with macrophages. Our previous research has already identified that MSCs and macrophages participated in the formation of the TME[22]. The results from Fig. 4H and Fig. S3B suggested that MSCs may play an immunomodulatory role in the microenvironment. Additionally, The involvement of senescent endothelial cells in the construction of the tumor immune microenvironment should not be overlooked, as there were literature reporting that senescent cells can recruit immunosuppressive macrophages, exacerbating the immunosuppressive tumor microenvironment by increasing the infiltration of regulatory T cells and impairing antitumor T cell immunity[53]. Whether senescent endothelial cells have similar physiological functions in the TME is a question that warrants particular consideration in future.

The characteristic of cellular senescence is enhanced transcription and protein synthesis, which leads to the release of SASP[29]. In order to illustrate the mechanism by which the senescent ECs recruit MSCs into TME, we further analyzed our sc-RNA seq data and noticed that the CD34^+^CLDN5^+^ECs may recruit the MSCs into the tumor tissue by IGF system. Recent studies suggest that IGF proteins are highly expressed by senescent cell and played a significant role in marrow-derived cellular recruitment. Yang *et al*. focused on IGF-binding proteins-rP1 from senescent fibroblasts, which regulated prostate tumor progression and angiogenesis in animal models[54]. Maeng *et al*. demonstrated that locally generated IGF2 at tumor sites recruited the marrow-derived endothelial progenitor cell into the microenvironment via IGF2/IGF2R axis[38]. Here, we illustrated that the potential effect of IGF2/IGF2R axis between the MSCs and CD34^+^CLDN5^+^ECs contributed to formation of CCA. In vitro, we noticed that the IGF2 enhanced the migratory ability of MSCs and was recruited by IGF2. However, when the expression of IGF2R in MSCs was silenced, the facilitated effect of IGF2 in MSCs was inhibited. Moreover, IGF system can activate the MSCs by activation of MAPK signaling and PI3K signaling[55], [56]. In our project, it was revealed that IGF2 activated the MAPK signaling in MSCs to enhance the migratory capacity rather than PI3K signaling. In vivo, the silent IGF2R MSCs were utilized to treat the D12 rats, found that this inhibited the increase of CCA, alleviated the tumor burden and injury of liver, which caused by MSCs. Meanwhile, MSCs were mainly enriched in paracancerous tissue and located far away from the CD34^+^CLDN5^+^ cells in cancerous tissue. In addition, we found that IGF2, as a member of SASP, was regulated by CEBPβ in senescent ECs, which specifically combined with *Igf2*-DNA-promoter sequence. Currently, there is a growing awareness of the role of the IGF signaling pathway in the TME. It has been found that IGF2 is overexpressed in various types of cancer, including breast, prostate, and colorectal cancers, and is associated with chemotherapy resistance and poor prognosis. Many researchers are actively developing small molecule drugs that inhibit the binding of IGF2 to its receptor, thereby blocking the signaling pathways that promote tumor growth and survival[57]. Our research described the regulatory role of IGF2 in the TME from a new perspective, targeting the IGF2/IGF2R/MAPK axis to potentially suppress the CCA in primary liver cancer. In the future, further methods to intervene in the IGF signaling pathway can be explored, offering possibilities for treatment of primary liver cancer.

**Fig. 8:**
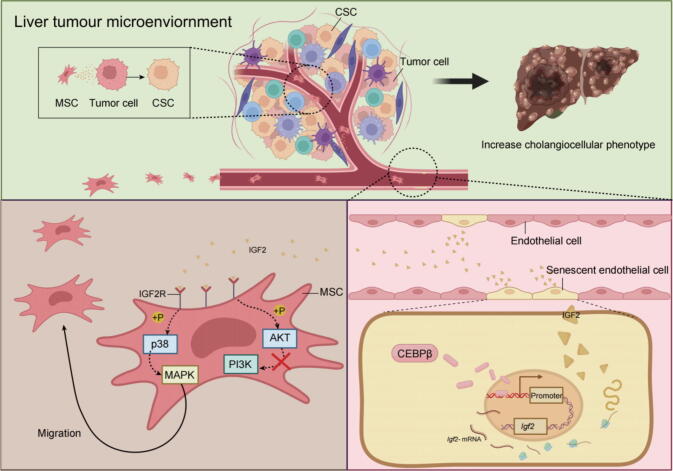
Tumor associated senescent endothelial cells recruited the mesenchymal stem cells into the TME, promoting the formation of cholangiocellular phenotype in HCC A schematic model of the mechanism by which CD34^+^CLDN5^+^ ECs, with distinct senescent cellular features and high expression of IGF2, which was regulated by CEBPβ, recruited the MSCs into TME via IGF2R/MAPK. Then, MSCs in TME released the cytokines to induced evolution of the HCC cell into CSC-like characteristic tumor cell, promoted the increasing of cholangiocellular phenotype and malignant transformation of HCC.

Our schematic model of the mechanism is shown in Fig. 8. In summary, we identified a novel subset of CD34^+^CLDN5^+^ECs, with distinct senescent cellular features and high expression of IGF2, which was regulated by CEBPβ, primarily existed in the HCC tumor tissue. Specifically, the ECs recruited MSCs into the TME through the IGF2/IGF2R/MAPK signaling pathway. Once within the TME, these MSCs secreted a profile of cytokines (including: CCL2, CCL5, HGF, TGF-β and GM-CSF). The release of these cytokines was instrumental in driving the evolution of HCC cells towards CSC-like characteristics, increasing malignancy and promoting CCA. Our findings also correlated with clinical samples. The CD34^+^CLDN5^+^ cells with senescent features not only predominantly exited in iCCA and cHCC-CCA patients, but also were related with the patients' survival duration of liver cancer. Clinically, a significant reason for the poor prognosis of iCCA and cHCC-CCA is the difficulty in early diagnosis and screening. Most patients are already in advanced stages when they are diagnosed, and the diagnosis often relies heavily on the extensive experience of clinical physicians or histopathological examinations[58]. Therefore, the CD34^+^CLDN5^+^ cells may serve as a potential clinical prognostic marker for HCC. These discoveries introduced the concept of senescent ECs in TME for the first time, allowing us to better understand the recruitment capacity of senescent cell and the mechanism underlying of promoting liver cancer.

Currently, the first-line treatment options for most advanced HCC patients include anti-angiogenic targeted drugs such as sorafenib and lenvatinib. However, the response rate of the advanced patients to these drugs is not ideal. Whether to use indiscriminately anti-angiogenic drugs to suppress tumors is a question worth considering. Recent studies support the idea that senolytics can reshape the TME, and the ability of senolytics (Quercetin) to specifically kill senescent endothelial cells, have caught our attention[59], [60]. The era is ripe for senolytics to be progressively advanced into clinical settings, and our research also provides a theoretical basis for the involvement of senescent cells in the construction of the TME. Whether the combination of senolytics and immune checkpoint inhibitors can become a new strategy for the treatment of primary liver cancer is a question that warrants further exploration[61].

## Compliance with Ethics Requirements

Each participant signed an informed consent form before participating in the study, which was approved by the Ethics Committee of the Third Affiliated Hospital of Naval Medical University (Number: EHBHKY2017-K-025).

All experiments of animals were conducted complied with the ethical policies approved by Naval Medical University Animal Care Committee (Number: 20175001123).

## CRediT authorship contribution statement

**Xin-yu Zhu:** Conceptualization, Methodology, Investigation, Writing – original draft, Validation, Formal analysis, Visualization, Project administration. **Wen-ting Liu:** Validation, Formal analysis, Visualization, Supervision, Project administration. **Xiao-juan Hou:** Validation, Formal analysis, Visualization, Supervision, Project administration. **Chen Zong:** Validation, Formal analysis, Visualization, Supervision, Project administration. **Wei Yu:** Validation, Formal analysis, Visualization. **Zhe-min Shen:** Resources, Data curation. **Shu-ping Qu:** Resources, Data curation. **Min Tao:** Resources, Data curation. **Meng-meng Xue:** Resources, Data curation. **Dao-yu Zhou:** Resources, Data curation. **Hao-ran Bai:** Resources, Data curation. **Lu Gao:** Funding acquisition. **Jing-hua Jiang:** Resources, Data curation. **Qiu-dong Zhao:** Resources, Data curation. **Li-xin Wei:** Supervision, Project administration, Resources, Writing – review & editing. **Xue Yang:** Supervision, Project administration, Resources, Writing – review & editing. **Zhi-peng Han:** Supervision, Project administration, Resources, Writing – review & editing. **Li Zhang:** Conceptualization, Methodology, Investigation, Writing – original draft, Formal analysis, Validation, Visualization, Resources, Writing – review & editing, Funding acquisition.

## Declaration of competing interest

The authors declare that they have no known competing financial interests or personal relationships that could have appeared to influence the work reported in this paper.
