# Supplementary material for: CD34+CLDN5+ tumor associated senescent endothelial cells through IGF2-IGF2R signaling increased cholangiocellular phenotype in hepatocellular carcinoma
Source: J Adv Res. 2024 Dec 12;76:511–28. doi: 10.1016/j.jare.2024.12.008 (PMC12793740; doi:10.1016/j.jare.2024.12.008)
Supplement: Supplementary Data 1 [file mmc1.docx]

CD34^+^CLDN5^+^ Tumor Associated Senescent Endothelial Cells through IGF2-IGF2R signaling Increased Cholangiocellular Phenotype in Hepatocellular Carcinoma

**Supplement figures**

**
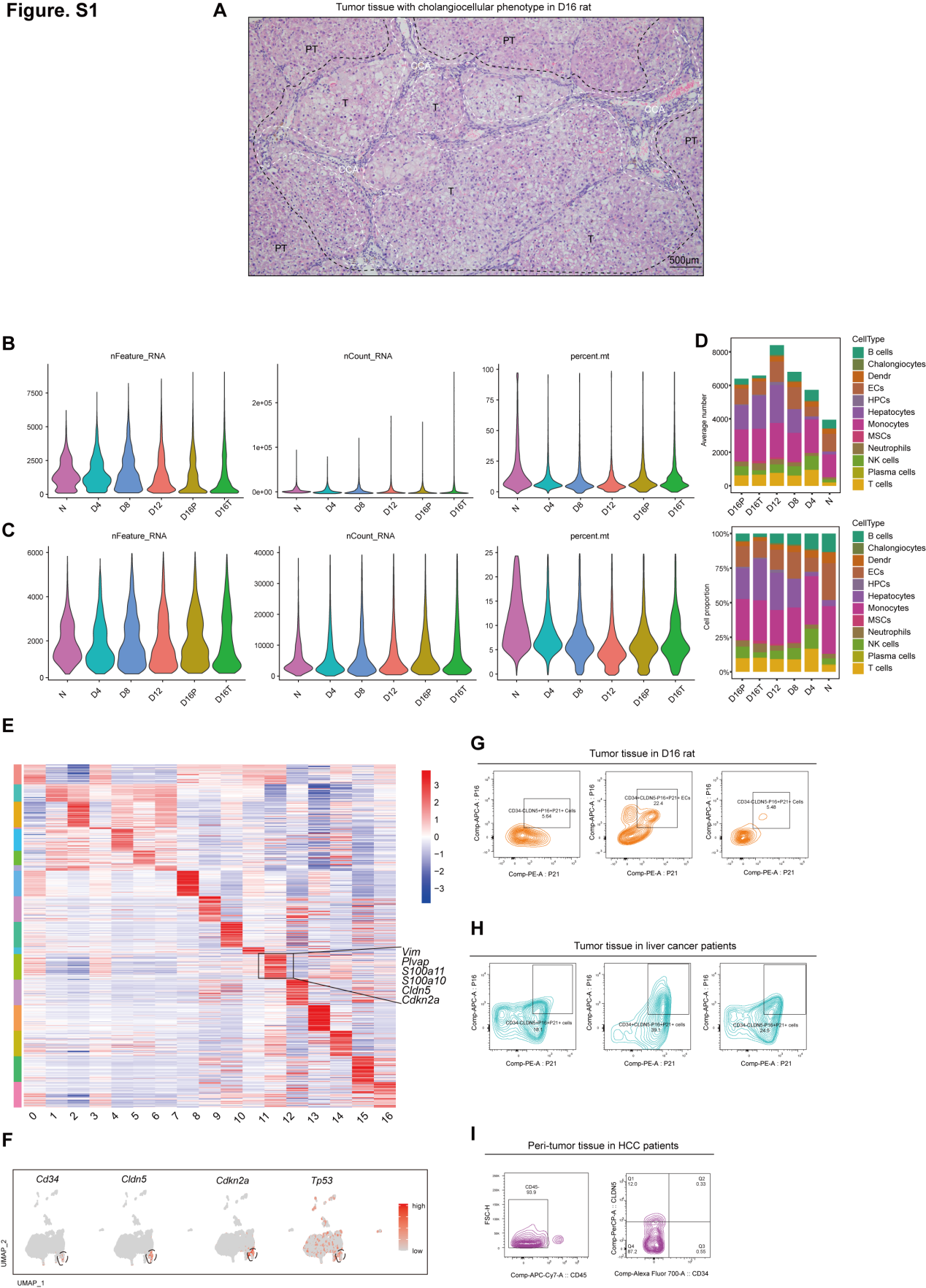
**

**Fig. S1**

**(A)** H&E staining of liver specimens from the different D16T. Scale bars, 100 μm. White Dotted portion shows the cholangiocarcinoma phenotype area in tumor tissue. Black Dotted portion shows the tumor area. PT, peri-tumor ; T, tumor; CP, cholangiocarcinoma phenotype. Scale bars, 500µm. (**B)** Violin plots showing the scRNA-seq original data of different samples before quality control. nFeature_RNA, the detected gene number in each cell; nCount_RNA, unique molecular identifier (UMI) in each cell; percent.mt, mitochondrial percentage of each cell. (**C)** Violin plots showing the scRNA-seq data of different samples after quality control. **(D)** The proportion and average number of the distinct cell types in different liver samples. **(E)** Heat map depicting expression levels of genes in different ECs subtypes, with the lowest expression levels represented as blue box and the highest expression levels as red box. Rows represent genes and columns represent clusters. Highlighting the cellular senescent-associated genes in Cluster 11. (**F)** The UMAP plot showing the projection of expression levels of *Cd34*, *Cldn5*, *Cdkn2a*, and *Tp53 in* ECs subtypes, with the lowest expression levels represented as gray dots and the highest expression levels as red dots. (**G)** D16 rats’ tumor tissue, n = 3: The P16^+^P21^+^cells proportion in CD34^+^CLDN5^-^ cells, CD34^-^CLDN5^-^ cells and CD34^-^CLDN5^+^ cells were measured by flow cytometry. **(H)** HCC patients’ tumor tissue, n = 2: The P16^+^P21^+^cells proportion in CD34^+^CLDN5^-^ cells, CD34^-^CLDN5^-^ cells and CD34^-^CLDN5^+^ cells were measured by flow cytometry. **(I)** HCC patients’ peri-tumor tissue, n=2: CD34^+^CLDN5^+^ cells proportion in non-parenchymal cells were measured by flow cytometry.


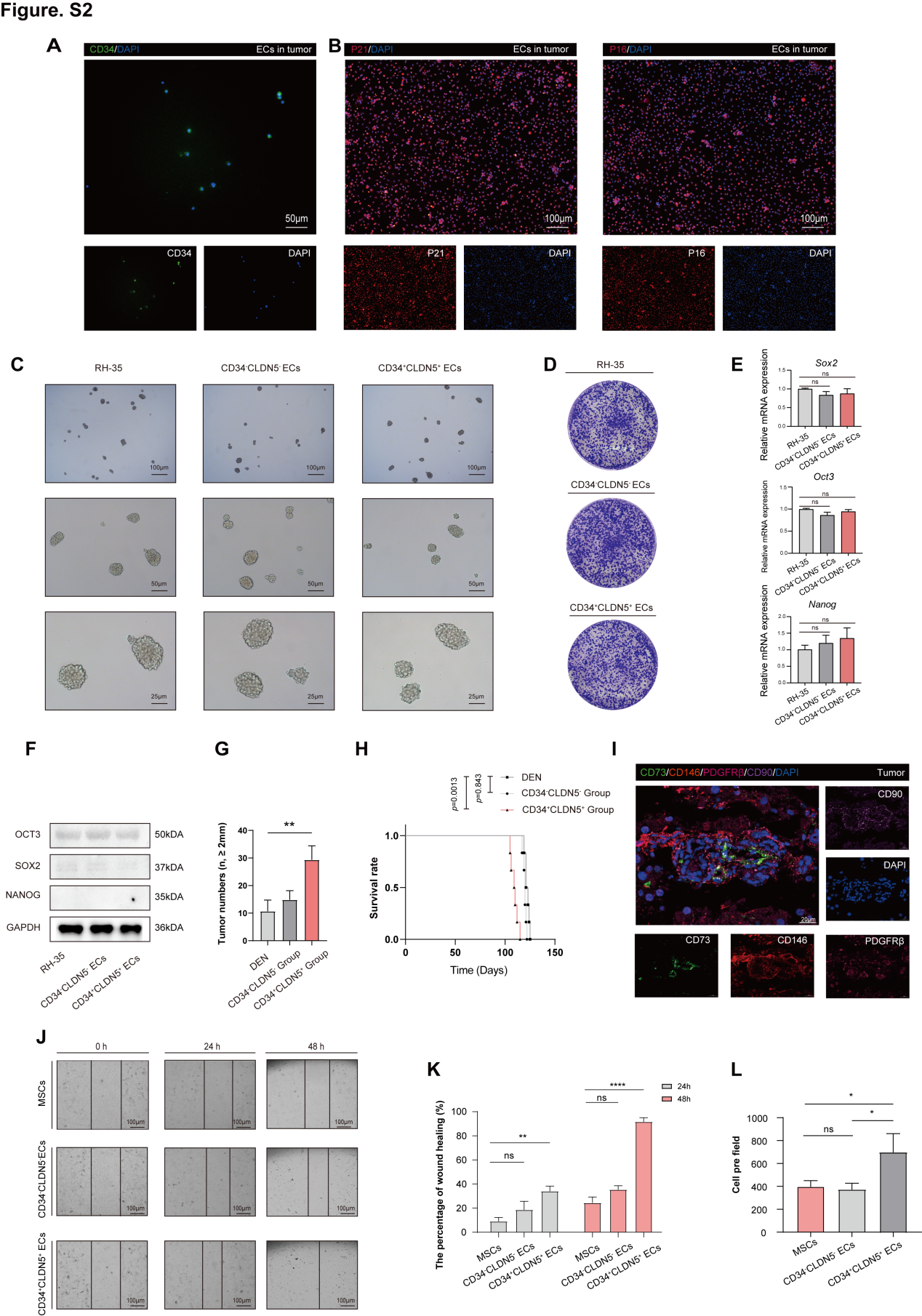


**Fig. S2**

**(A)** Characterized the CD34^+^CLDN5^+^ ECs from D16 rats by Immunofluorescence staining of CD34. Scale bars, 50 μm. (**B)** Immunofluorescence staining of P21 and P16 in CD34^+^CLDN5^+^ ECs. Scale bars, 100 μm. (**C)** Sphere ability of RH-35 cells which co-cultured with different ECs were examined using sphere formation assay. Scale bars, 100 μm, 50 μm, 25 μm. (**D)** Clonogenic ability of RH-35 cells which co-cultured with different ECs were examined using colony formation assay. (**E)** Expression of stemness-related mRNA which folded over *Gapdh* was examined by RT-PCR in RH-35 which co-cultured with different ECs. Data are represented as mean ± SD. ns, no significant. (**F)** Expression of stemness-related protein was examined by WB in RH-35 which co-cultured with different ECs. (**G)** The number of tumor nodules (Diameter ≥2mm) per liver in D16 rats. Data are represented as mean±SD. **p < 0.01. n=6, respectively. (**H)** The Kaplan-Meier overall survival curves of rats exposed to DEN. p value was determined by Kaplan-Meier survival curves and log-rank test. n=6, respectively. (**I)** Multi-color staining by IHC of CD73, CD90, CD146 and PDGFRβ in liver specimens from the D16T. Scale bars, 20 μm. (**J)** Wound healing assay for rat primary MSCs which co-cultured with the different ECs for 0 h, 24 h and 48 h. The black main strings show the wound area. Scale bars, 100 μm. (**K-L)** Quantified data of transwell migration assay and wound healing assay of primary MSCs which co-cultured with the different ECs. Data are represented as mean ± SD. **p* < 0.05, ***p* < 0.01, *****p* < 0.0001 ; ns, not significant.


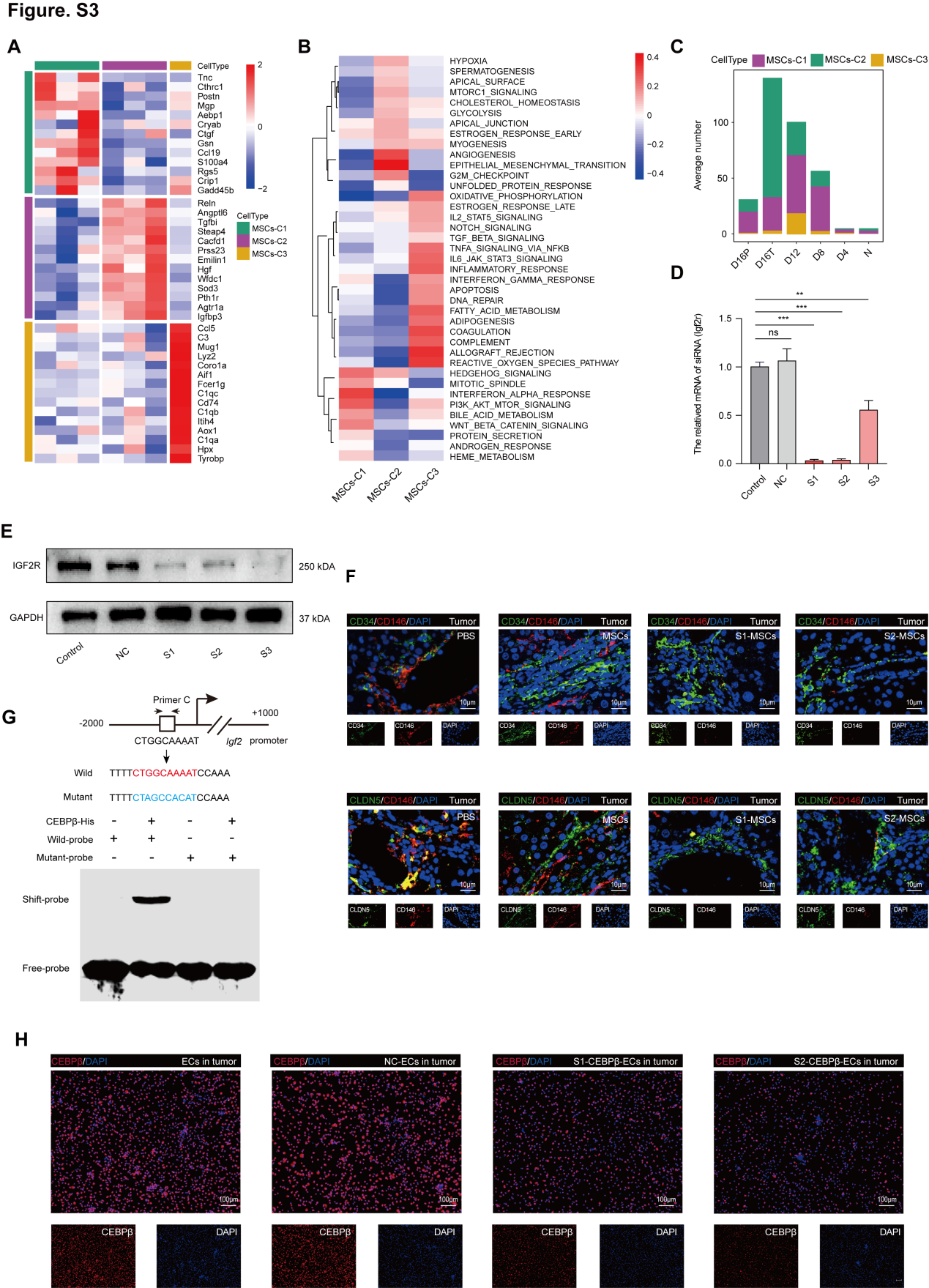


**Fig. S3**

**(A)** Heat map of the characteristic markers of each cluster in the MSC subtypes. (**B)** Heatmap of the different MSCs subtypes functional enrichment analysis. **(C)** The average number of the different MSCs subtypes in different liver sample. (**D)** Expression of *Igf2r* mRNA was examined by RT-PCR in rat primary MSCs treated with *Igf2*r-siRNA (100 nM) or scramble siRNA (as negative control) or not (as Control) for 48 h. Data are represented as mean±SD. Significance between control and other groups was performed using Student’s t test. ***p* < 0.01, ****p* < 0.001. (**E)** Expression of IGF2R was examined by WB in rat primary MSCs treated with *Igf2*r-siRNA (100 nM) or scramble siRNA (as negative control) or not (as Control) for 48 h. ns, negative control; S1, *Igf2*r-siRNA candidate 1; S1, *Igf2*r-siRNA candidate 2; S3, *Igf2*r-siRNA candidate 3; NC, negative control. **(F)** Immunofluorescence staining staining with CD34, CLDN5 and CD146 to the different liver specimens from the D16T. PBS, the rats were administrated by PBS; MSCs, the rats were administrated by MSCs; MSCs-S1, the rats were administrated by MSCs which treated with *Igf2*r-siRNA candidate 1; MSCs-S2, the rats were administrated by MSCs which treated with *Igf2*r-siRNA candidate 2. Scale bars, 10 μm. (**G)** Competitive EMSA to detect the binding of CEBPβ to promoters of *Igf2*. Wild: Labeled probes sequence; Mutant: Mutant probes sequence; The bands at the upper and lower part of membranes indicate shift (protein-probe complex) and unbound free probes, respectively. (**H)** Immunofluorescence staining of CEBPβ for CD34^+^CLDN5^+^ECs treated with *Cebp**β*-siRNA (100 nM) or scramble siRNA (as negative control) or not (as Control) for 48 h. S1-CEBPβ-ECs, CD34^+^CLDN5^+^ECs which treated with *Cebpβ*-siRNA candidate 1; S2-CEBPβ-ECs, CD34^+^CLDN5^+^ECs which treated with *Cebpβ*-siRNA candidate 2; NC, negative control. Scale bars, 100 μm.


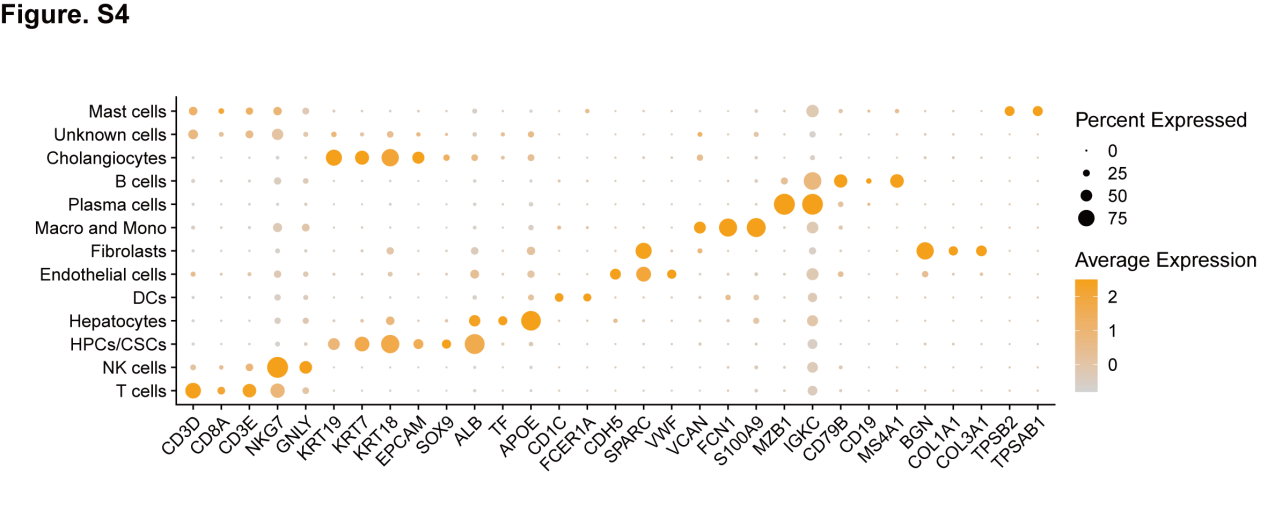


**Fig. S4**

The process of categorizing and distinguishing cells based on their distinct marker gene expressions.

**Supplement table**

**Table S1. Demographic and baseline characteristics of 68 HCC patients**

| **Factors** | **Value** | **Percent [%]** |
| --- | --- | --- |
| Age | 56.38 ± 11.51 |  |
| Gender (male/female) | 48/20 | 70.59/29.41 |
| HbsAg (positive) | 35/33 | 51.47/48.53 |
| Maximum tumor diameter | 6.85 ± 3.33 |  |
| Tumor number (multiple) | 12 | 17.65 |
| Tumor thrombosis | 5 | 7.35 |
| Alpha fetoprotein (ng/mL) | 98.13 ± 266.28 |  |
| Total bilirubin (μmol/L) | 18.16 ± 24.39 |  |

**Table S2. Sequences for *Igf2r* siRNA**

| **Product name** | **Forward sequence (5’-3’)** | **Reverse sequence (5’-3’)** |
| --- | --- | --- |
| siRNA candidate 1 | GGAUAAGAGUGGAAGUAAATT | UUUACUUCCACUCUUAUCCTT |
| siRNA candidate 2 | GCAUGGUGUCAGAAGACAATT | UUGUCUUCUGACACCAUGCTT |
| siRNA candidate 3 | CGAAGAAGAUGGAGUGCAATT | UUGCACUCCAUCUUCUUCGTT |
| siRNA NC | UUCUCCGAACGUGUCACGUTT | ACGUGACACGUUCGGAGAATT |

**Table S3. Sequences for *Cebpβ* siRNA**

| **Product name** | **Forward sequence (5’-3’)** | **Reverse sequence (5’-3’)** |
| --- | --- | --- |
| siRNA candidate 1 | GGCCCUGAGUAAUCACUUATT | UAAGUGAUUACUCAGGGCCTT |
| siRNA candidate 2 | CCGUUUCGAGCAUUAAAGUTT | ACUUUAAUGCUCGAAACGGTT |
| siRNA NC | UUCUCCGAACGUGUCACGUTT | ACGUGACACGUUCGGAGAATT |

**Table S4. Primers for CUT&Tag qPCR analysis**

| **Product name** | **Forward sequence (5’-3’)** | **Reverse sequence (5’-3’)** |
| --- | --- | --- |
| Primer A | CATCAATACCAGCGAGAGCCA | GTAGCGGCCTTCTCAATTCCT |
| Primer B | AGCAACTTCGATTGGAACCAC | ACCTAGGGGTCAGGCTACTC |
| Primer C | CCTCTTGGGTCCCCCAATTT | TTAGGTTTGCAAGCGTTAACAGG |

**Table S5. Primers for RT-qPCR analysis**

| **Product name** | **Forward sequence (5’-3’)** | **Reverse sequence (5’-3’)** |
| --- | --- | --- |
| Rat*-Nanog* | AACGCTGCTCCGCTCCATAAC | CTGGCTTTCCCTAGTGGCTTCC |
| Rat-*Oct3* | GGACTAGCATTGAGAACCGTGTGAG | ACATCCCTCTCCAGCCCAAGC |
| Rat-*Sox2* | CCTCTTGGGTCCCCCAATTT | TTAGGTTTGCAAGCGTTAACAGG |
| Rat-*Igf2r* | TGGCTCGTCACTCAGAATCA | CGGCAGCACCTTCAGGACAG |
| Rat-*Igf1r* | AACGCTTCAGTTCCTTCCATTCCTC | CCGCTGCCACCTCACAATGTAG |
| Rat-*Insr* | atccgtcgctcctatgctctgg | TGATCGTGAGGTTGTGCTTGTTCC |
| Rat-*Gapdh* | caagttcaacggcacagtcaagg | acatgctcagcaccagcatcacc |
